# Development of CD33-Targeted Dual Drug-Loaded Nanoparticles for the Treatment of Pediatric Acute Myeloid Leukemia

**DOI:** 10.1021/acs.biomac.4c00672

**Published:** 2024-09-05

**Authors:** Ana M. Carvalho, Michelle K. Greene, Peter Smyth, Alexander Mutch, Kirsty M. McLaughlin, Lauren V. Cairns, Ken I. Mills, Karen D. McCloskey, Christopher J. Scott

**Affiliations:** The Patrick G Johnston Centre for Cancer Research, School of Medicine, Dentistry and Biomedical Sciences, Queen’s University Belfast, Belfast BT9 7AE, U.K.

## Abstract

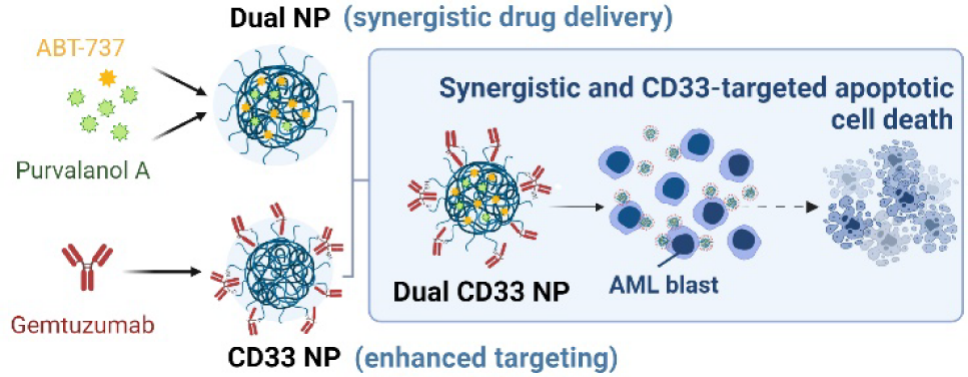

Paediatric acute myeloid leukemia (AML) is a heterogeneous hematological malignancy still heavily reliant on traditional chemotherapeutic approaches. Combination treatments have shown to be a superior approach, but their success is often hindered by side effects and different drugs’ pharmacokinetics. Here, we investigated ABT-737 and Purvalanol A as a potential drug pairing for pediatric AML and described the development of CD33-targeted polymeric nanoparticles (NPs) to enable their simultaneous targeted codelivery. Separate drug encapsulation within poly(lactic-*co*-glycolic acid) (PLGA) NPs was optimized prior to coencapsulation of both drugs at a synergistic ratio in PEGylated PLGA NPs. The therapeutic effects of formulations were evaluated in a panel of pediatric AML cells, and dual drug-loaded NPs (dual NPs) demonstrated significantly enhanced apoptotic cell death. Moreover, conjugation to gemtuzumab resulted in improved NP binding and internalization in CD33-positive cells. Finally, CD33-targeted dual-loaded NPs showed enhanced cytotoxicity to CD33-positive AML cells via CD33-mediated targeted drug delivery.

## Introduction

Accounting for approximately 20% of childhood leukemia cases, acute myeloid leukemia (AML) is responsible for the majority of leukemia-related deaths.^1,2^ While there has been great improvement in the understanding of AML pathophysiology and approval of new drugs,^3^ the prognosis and treatment of pediatric AML remain challenging due to high relapse rates, disease heterogeneity, and therapy-related toxicity.^4^

Precision medicine has been gaining importance in the treatment of AML, leading to the emergence of novel targeted therapies and the approval of 10 new therapies by the U.S. Food and Drug Administration (FDA) in the past few years.^3,5^ These range from mutation-specific therapies, targeting common genetic lesions in AML such as mutant fms-like tyrosine kinase 3 (FLT3) with drugs like midostaurin or quizartinib, to therapies targeting the apoptotic pathway such as the Bcl-2 inhibitor venetoclax or antibody drug conjugates (ADCs) like gemtuzumab ozogamicin (GO).^6,7^ GO consists of a monoclonal antibody (mAb) targeting CD33 (gemtuzumab) covalently attached to a toxin (calicheamicin) and is currently the only targeted therapy approved by the FDA for pediatric AML.^8,9^ The CD33 transmembrane protein is a well-known target for AML, being heavily expressed on the cell surface of malignant AML blasts with high expression levels correlated with poor disease prognosis.^10^

Despite the development of targeted drugs, combination drug cocktails can confer significant advantages and improved efficacy over monotherapies. However, success is often hindered by the undesired side effects and different pharmacokinetic profiles of the drugs.^11^ The development of drug-loaded nanosystems offers a promising means to overcome the main issues of current combination therapy, whereby multiple drugs may be entrapped within a single nanocarrier. The clinically established success of these nanosystems is perhaps best illustrated by CPX-351, a liposomal formulation coencapsulating daunorubicin and cytarabine at a synergistic 1:5 molar ratio, which is currently approved for AML treatment.^12^ The use of such nanoparticle (NP)-based drug carriers can not only reduce adverse off-site effects but also improve pharmacokinetics and offer ratiometric drug delivery.^13^

In this current work, we evaluated the coadministration effects of ABT-737 and Purvalanol A, which have been shown to elicit therapeutic effects toward pediatric AML harboring both mixed lineage leukemia (MLL) rearrangement and internal tandem duplications (ITDs) of the FLT3 gene.^14^ ABT-737 is a small molecule BH3 mimetic that binds and inhibits the antiapoptotic B-cell lymphoma (Bcl) proteins Bcl-2, Bcl-w, and Bcl-xL, often upregulated in cancer cells and associated with tumor resistance.^15,16^ As a single agent, ABT-737 has shown significant potency against leukemia cells, but therapeutic potential was compromised by its poor solubility and bioavailability, as well as dose-dependent thrombocytopenia.^17−19^ Purvalanol A acts as a cyclin-dependent kinase (CDK) inhibitor and exerts its function by blocking the binding of CDK1 or CDK2 with their specific cyclin counterparts. This interaction leads to cell cycle arrest, preventing uncontrolled proliferation and inducing apoptosis in cancer cells.^20−22^

Based on our previous experience with encapsulation of drugs in poly(lactic-*co*-glycolic acid) (PLGA)-based NPs,^16,23−27^ we devised the development of a novel dual-drug CD33-targeted delivery system based on PEGylated PLGA NPs to coencapsulate and deliver both drugs at a synergistic ratio. Following development and nanoformulation characterization, we examined its therapeutic efficacy in CD33-positive pediatric AML cell models.

## Experimental Section

### Nanoparticle Formulation

NPs were prepared using the single emulsion solvent evaporation method previously described.^16,26^ Unless stated otherwise, each NP batch contained 20 mg of polymer (either PLGA RG502H (Sigma-Aldrich) alone or different blends of PLGA RG502H with PLGA-PEG-maleimide (Creative PEGworks; *M*_w_ ∼ 20 000:5 000 Da)). For dual-loaded NP development, 40 μg of ABT-737 (Selleckchem) at 10 mM in DMSO and 900 μg of Purvalanol A (Selleckchem) at 50 mM in DMSO were added to the organic phase. Alternatively, fluorescent NPs were developed for binding assays by replacing the drugs with 200 μg of rhodamine 6G prepared at 2 mg/mL in DCM. NP batches were divided into four vials containing 5 mg of polymer each and washed by three wash-spin cycles (17 000*g*, 20 min, 4 °C). Between centrifugations, each NP pellet was resuspended in 1 mL of 55 mM SDS (for the first wash) or 1 mL of PBS (for the remaining two washes) via pulsed probe sonication with a Model 120 Sonic Dismembrator (Fisher Scientific), in cycles of 1 s on followed by 1 s off at 30% amplitude.

### Antibody–Nanoparticle Conjugation

For conjugation, 25 μg of gemtuzumab (hP67.6, Absolute Antibody) was pretreated with 40× molar excess of tris(2-carboxyethyl)phosphine hydrochloride (TCEP HCl) (Fluorochem) at 0.1 mg/mL in PBS with 1 mM ethylenediaminetetraacetic acid (EDTA) for 3 h at 37 °C while shaking at 300 rpm. The reduced antibody solution was purified using a Zeba Spin desalting column (Thermo Fisher Scientific, 7 kDa, 0.5 mL) as per manufacturer’s instructions, prior to addition to 1 mg of PLGA-PEG-maleimide nanoparticles resuspended in PBS at 1 mg polymer/mL and allowed to slowly rotate overnight at 4 °C. The next day, nanoparticles were collected by centrifugation (12 000*g*, for 20 min at 4 °C) and washed twice with PBS in resuspension and centrifugation cycles under the same conditions.

### Characterization of Nanoparticles

Nanoparticle hydrodynamic diameter, zeta potential, and polydispersity index (PdI) were analyzed using a NanoBrook Omni (Brookhaven Instruments) upon resuspension of the samples via sonication in 2% (v/v) PBS in dH_2_O at a concentration of 0.1 mg polymer/mL. Further characterization of nanoparticle size was performed via nanoparticle tracking analysis (NTA) using a Nanosight NS300 (Malvern) with nanoparticles suspended in PBS at 0.1 mg polymer/mL. Transmission electron microscopy (TEM) was carried out on a JEM 1400plus electron microscope (JEOL) operating at 80 kV for assessment of nanoparticle morphology. To quantify ABT-737 and Purvalanol A encapsulation, blank nude NPs were dissolved at 5 mg polymer/mL in a 1:1 mixture of acetonitrile (ACN): dimethyl sulfoxide (DMSO) and used as a diluent to construct ABT-737 and Purvalanol A standard curves (Figure S1). These were transferred to a UV-Star clear 96-well microplate together with the NP samples dissolved in the same manner, and absorbance was read at 340 and 440 nm, respectively, for Purvalanol A and ABT-737. For quantification of conjugated gemtuzumab, nanoparticles were resuspended at 2 mg polymer/mL in PBS and a Micro Bicinchoninic Acid (Micro BCA) Protein Assay Kit (Thermo Scientific) was used according to the manufacturer’s instructions, using blank nude NPs as the diluent for the standard curve creation.

### *In Vitro* Drug Release Studies

To study the release profile of the drugs from the nanoformulations, dual NPs were resuspended in PBS with 10% (v/v) fetal bovine serum (FBS) at 1 mg polymer/mL and equally distributed between several 2 mL Eppendorf vials (1.5 mL/vial). The vials were kept under constant shaking (200 rpm) at 37 °C and at various time points (0, 15, and 30 min, and 1, 3, 8, 24, and 48 h), one vial aliquot was taken out, centrifuged at 16 000*g* for 20 min, and washed once with SDS and twice with PBS to remove any unencapsulated drug. The drug content in the washed nanoparticle pellets was then quantified as previously described (see Characterization of Nanoparticles). Finally, the amount of drug released from the nanoparticles at each time point (*t* = *x*) was calculated with respect to the initial amount of drug loaded (time point of *t* = 0 h), according to the equation below.
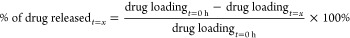


### Stability Studies

To evaluate the stability profile of the developed nanoformulations, dual NP pellets were stored at −20 °C, 4 °C, and room temperature (RT) for increasing amounts of time (0, 1, 4, 7, 14, and 21 days). At each time point, NPs were washed and characterized in terms of size, PdI, and zeta potential (measured using the NanoBrook Omni from Brookhaven Instruments), as well as drug encapsulation.

### SDS-PAGE Gel Electrophoresis

A 10% glycine-based acrylamide gel was used to run the samples together with an MW marker (PageRuler Plus Prestained Protein Ladder, Thermo Fisher Scientific). 5 μg portion of antibody samples or 0.5 mg of NPs was prepared in 20 μL of PBS, mixed with 5 μL of nonreducing 5× loading buffer, heated at 95 °C for 10 min, and loaded into the wells of the gel. Electrophoresis was carried out at 100 V for 20 min and then at 120 V for another 50–70 min until appropriate protein separation was achieved. Finally, gels were stained with InstantBlue Coomassie Protein Stain (Abcam) and washed with dH_2_O prior to imaging.

### Fluorescence-Linked Immunosorbent Assay (FLISA)

FLISA studies were performed as previously described,^26^ using recombinant human CD33-Fc (Sino Biological) at 1 μg/mL to coat the plate wells. All concentrations of unconjugated or gemtuzumab-conjugated rhodamine 6G-loaded nanoparticles, CD33-Fc, and free gemtuzumab are indicated in the figure legends. For targeting specificity studies, several modifications were made to the protocol, namely: (1) NPs were preincubated with CD33-Fc antigen for 30 min at room temperature, prior to their addition to the plate; (2) 100 μL of free gemtuzumab at 40 μg/mL in blocking buffer was added to the wells of the antigen-coated plate for 2 h at room temperature, prior to plate washing three times, with 300 μL of wash buffer per well, and addition of NPs; (3) NPs were premixed with free gemtuzumab at varying concentrations (0.00256–40 μg/mL) prior to addition of the mixture to the plate.

### Surface Plasmon Resonance (SPR)

Nanoparticle binding was assessed on a Biacore 8K instrument (GE Healthcare), and the experiments were performed in HBS-EP running buffer (Cytiva) at 25 °C. At first, a carboxymethylated dextran CM5 sensor chip (Cytiva) was activated with 0.4 M EDC and 0.1 M NHS. The chip was then functionalized with 15 μg/mL of recombinant human CD33-Fc (Sino Biological) or a control IgG1-Fc (R&D Systems) in 10 mM sodium acetate buffer at pH 5. Following this, 1 M ethanolamine hydrochloride at pH 8.5 was used to quench remaining NHS ester groups on the chip surface. These activation, functionalization, and quenching steps were performed at a constant flow rate of 10 μL/min, and 7 min of chip contact time were allowed for each step. Thereafter, nanoparticle suspensions prepared in HBS-EP buffer at the required concentrations (4, 2, 1, 0.5, and 0.25 mg polymer/mL) were injected over the coated chip at 20 μL/min for 15 s. Bound nanoparticles were then allowed to dissociate for 300 s, and the chip surface was regenerated between sample injections with three sequential treatment cycles of 50 mM sodium hydroxide at 30 μL/min for 30 s. Data are presented as response relative to baseline observed 5 s before the end of the injection period.

### Cell Culture

MV4-11, MOLM-13, and MOLM-14 pediatric acute myeloid leukemia cell lines were donated by Professor Ken Mills’ group (Patrick G Johnston Centre for Cancer Research, UK). Raji cells were obtained from the American Type Culture Collection. All cell lines were cultured in RPMI 1640 (Thermo Fisher Scientific) supplemented with 10% FBS and 1% penicillin–streptomycin (Thermo Fisher Scientific) and maintained at 5% CO_2_ and 37 °C in a humidified incubator (Sanyo Electrical Co., Ltd.).

### Cell Viability Assessment

Cell viability was evaluated using CellTiter-Glo luminescent assay (Promega) in accordance with the manufacturer’s instructions and expressed as a percentage of untreated control cells. Caspase activity was measured using the Caspase-Glo 3/7 assay (Promega) in accordance with the manufacturer’s instructions, and the data were expressed as a fold change relative to untreated control cells. Apoptotic cell death was also measured by Annexin V/PI staining. Following treatment, cell pellets were resuspended in 500 μL of 1× Annexin V Binding Buffer (BD Pharmingen), and 3 μL of Annexin V-FITC (BD Pharmingen) was added to the samples. After 15 min of incubation at room temperature, 2 μL of PI (Sigma) was added, and flow cytometry analysis was immediately performed using an Accuri C6 (BD Biosciences), recording a minimum of 10 000 events per sample.

### Cell Cycle Progression

Following treatment, the cells were collected and fixed by resuspending in 1 mL of ice-cold PBS/1% FBS followed by the addition of 4 mL of ice-cold absolute ethanol added dropwise while vortexing. The samples were then stored at 4 °C overnight, before washing with PBS and resuspended in 500 μL of FxCycle PI/RNase staining solution (Thermo Fisher Scientific) for 30 min at room temperature in the dark. Flow cytometry analysis was then carried out using an Accuri C6 (BD Biosciences), recording a minimum of 10 000 events per sample.

### Measurement of NP Binding to the Cell Surface

250 000 cells in 625 μL of cold media were added per microcentrifuge tube and allowed to chill for 15 min at 4 °C before addition of 375 μL of either cold PBS (for the controls) or NPs at 2 mg polymer/mL in PBS. Samples were incubated for 30 min under slow rotation in an orbital rotator at 4 °C and then kept stationary for a further 30 min at 4 °C. After this, the cells were collected and washed with wash buffer (PBS with 5% FBS (v/v)). For the staining of unoccupied CD33 receptors on the cell surface, 100 μL of staining solution containing 2.5 μL of PE-labeled mouse IgG1 isotype control (BioLegend) or PE-labeled antihuman CD33 antibody (BioLegend) in wash buffer was added to the cells and left to incubate for 30 min at 4 °C in the dark. Cells were then washed another three times and resuspended in 500 μL of wash buffer prior to flow cytometry analysis of PE-fluorescence using an Accuri C6 Flow Cytometer (BD Biosciences), recording a minimum of 10 000 events per sample.

### Confocal Microscopy Assays

For binding and internalization evaluation, 500 000 MOLM-13 cells were suspended in 0.9 mL of media in microcentrifuge tubes and cooled for 15 min in the fridge before 100 μL of treatments was added. Treatments included PBS (untreated control) and rhodamine-6G loaded NPs at 5 mg polymer/mL in PBS. Samples were incubated for 30 min under slow rotation in an orbital rotator at 4 °C and then kept stationary for a further 30 min at 4 °C. The cells were then collected and washed with cold PBS, before being resuspended in fresh media and transferred into a 12-well plate to allow internalization for 2 h at 37 °C. For the time course internalization study, the same amount of cells were seeded in 12-well plates, and 100 μL of rhodamine-6G loaded NPs in PBS at 2.5 mg polymer/mL was added to each well and incubated at 37 °C for different periods of time, including 30 min, 1 h, and 3 h. For both studies, cells were then collected into microcentrifuge tubes and washed by centrifugation and resuspension cycles as follows: once with cold PBS, once with cold acid strip buffer (50 mM glycine, 150 mM NaCl in PBS, pH 3), and again with PBS. The washed cells were then resuspended in 500 μL of cold PBS, and 100 μL of the cell suspension was spotted on a glass slide (VWR) via centrifugation at 400 rpm for 5 min in a Cytospin centrifuge. The cells were fixed on the slide by the addition of 4% (w/v) paraformaldehyde in PBS for 30 min in the dark and then washed once with PBS. Thereafter, cells were permeabilized with 0.5% (v/v) Triton X-100 in PBS for 10 min at room temperature and washed with PBS another three times. Finally, a drop of Vectashield Antifade Mounting Medium with DAPI (Vector Laboratories) was added, and the coverslip was carefully placed on top. Imaging was performed using a Leica SP8 confocal microscope (Leica, UK) or a Leica Stellaris microscope (Leica, UK), and images were obtained with a HCX PL APO 1.3NA/40× oil immersion objective zoomed 1× to 4× with a 1024 × 1024 frame and 400 Hz scan speed. Fluorescent images were obtained post excitation with a UV emitting diode (405 nm) and argon (488 nm), DPSS (561 nm), or HeNe (543, 594, and 633 nm) lasers as required. Images presented in the same panels were acquired by using standardized settings and parameters. Image analysis was conducted using the Leica LAS X software.

### Data Analysis

GraphPad Prism software (version 10.1.0) was used to graph data and perform statistical analyses. Student’s *t*-test was used to determine statistical significance in data sets comprised of two groups, and analysis of variance (ANOVA) with Tukey’s post hoc test was used to determine statistical significance in data sets of several groups. Statistical significance was defined as follows: *p < 0.05, **p < 0.01, ***p < 0.001, ****p < 0.0001. Synergy between drugs was evaluated by calculating their combination index (CI), determined according to the Chou–Talalay method,^28^ using the CompuSyn software. According to the method, CI = 1 indicates an additive combination, CI > 1 indicates an antagonistic effect, and CI < 1 indicates a synergistic effect. FlowJo software (version 10.8.1) was used to present flow cytometry histograms. Different NP batches were used to perform each replicate of the functional assays (binding, internalization, and cytotoxicity studies), so that batch variability is accounted for within the presented error bars.

## Results and Discussion

### Combination of ABT-737 and Purvalanol A Exhibits Synergistic Effects in Pediatric AML Cell Lines

**Figure 1:**
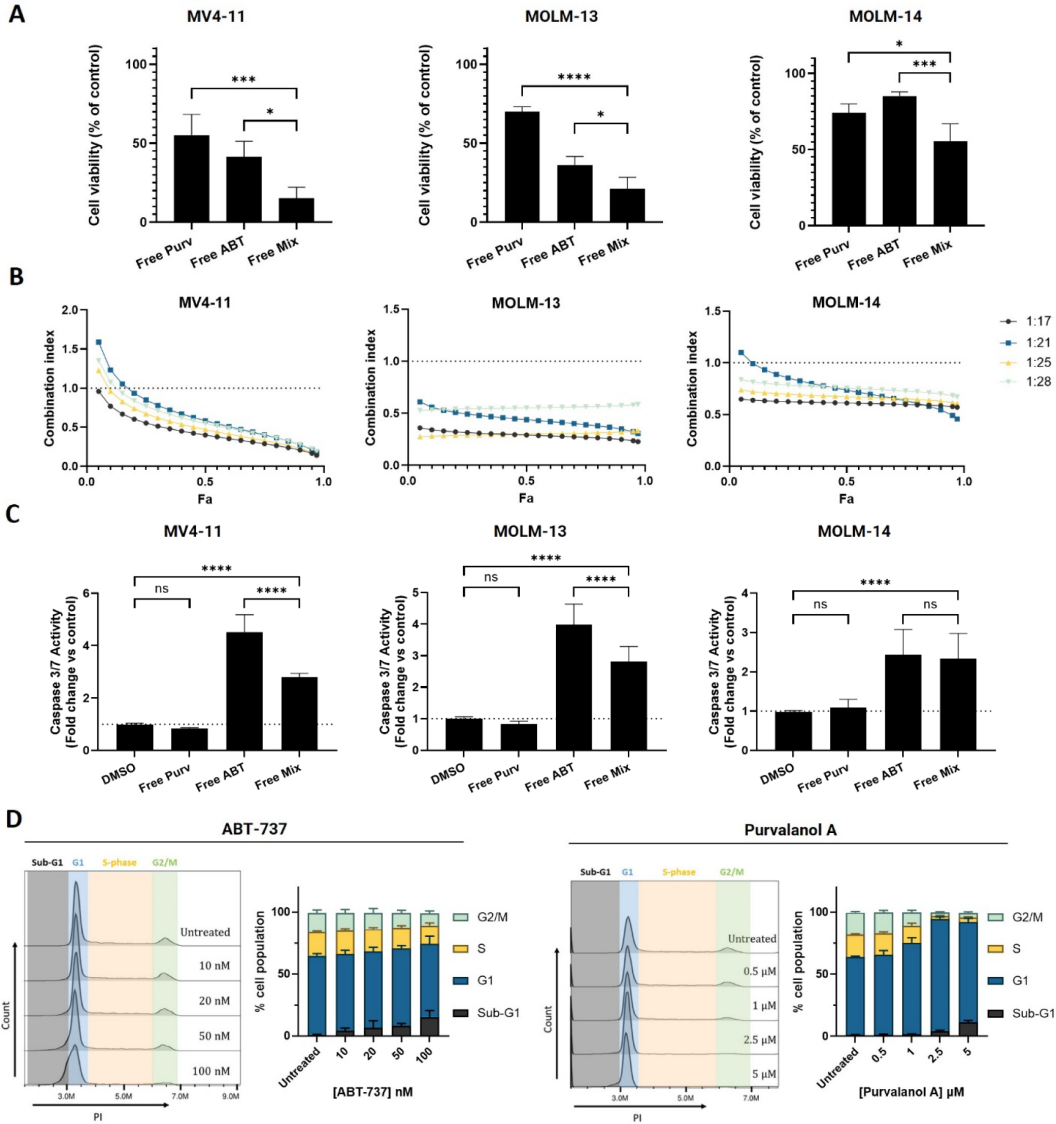
Effects of ABT-737 and Purvalanol A treatments on pediatric acute myeloid leukemia cell lines as separate and combined treatments. (A) MV4-11, MOLM-13, and MOLM-14 cells were treated for 72 h with 50 nM ABT-737 or 1.3 μM Purvalanol A, as single or mixed drug treatments, prior to assessment of cell viability by CellTiter-Glo. (B) Combination index (CI) values obtained after a 72 h drug cotreatment for each fraction of cells affected by the combination (Fa). CI values were calculated following CellTiter-Glo assay, using the Chou–Talalay method on CompuSyn software, where CI = 1 indicates an additive combination, CI > 1 an antagonistic effect, and CI < 1 a synergistic effect. Figure keys denote ABT-737/Purvalanol A molar ratios. (C) MV4-11, MOLM-13, and MOLM-14 cells were treated for 72 h with 50 nM ABT-737 or 1.3 μM Purvalanol A, as single or mixed drug treatments, prior to assessment of caspase 3/7 activity by Caspase 3/7 Glo. (D) Cell cycle analysis in MV4-11 cells using RNase/PI staining and flow cytometry upon 24 h of treatment with a range of concentrations of free ABT-737 or Purvalanol A. Data are presented as mean ± SD, *n* = 3.

An initial evaluation of the separate activity of ABT-737 and Purvalanol A was performed in a panel of pediatric AML cell lines carrying both MLL rearrangement and FLT3-ITD mutations (Figure S2). To assess the potential for synergy between ABT-737 and Purvalanol A, the effects of cotreatment with both agents were assessed at concentrations near their IC_50_ values determined across the three cell lines, and their combined cytotoxic effect was significantly greater than the effect of the separate drugs in all cell lines (Figure 1A). Moreover, synergistic cytotoxic effects were observed in all three cell lines, as indicated by combination indices <1, across a broad range of concentrations and molar ratios tested (Figure 1B). These findings are in agreement with other studies that similarly showed synergistic effects with the combination of Bcl-2 and CDK inhibitors.^29−31^ To better understand the mode of cell death, executioner caspase activation was examined, which showed that the combination significantly increased caspase 3/7 activation relative to the control, although interestingly, this effect was more pronounced with ABT-737 alone in MV4-11 and MOLM-13 cells (Figure 1C). This may be due to the contribution of Purvalanol A, which at the concentration tested is mainly responsible for cell cycle arrest in G1 phase, contrary to ABT-737, which directly contributes to apoptosis, as corroborated by the dose-dependent increase in sub-G1 population of cells (Figure 1D). These effects are consistent with recent observations that the combination of ABT-737 and Purvalanol A can activate intrinsic apoptotic mechanisms in AML cells.^32^

### Preparation and Characterization of Drug-Loaded PLGA-Based Nanoparticles

**Figure 2:**
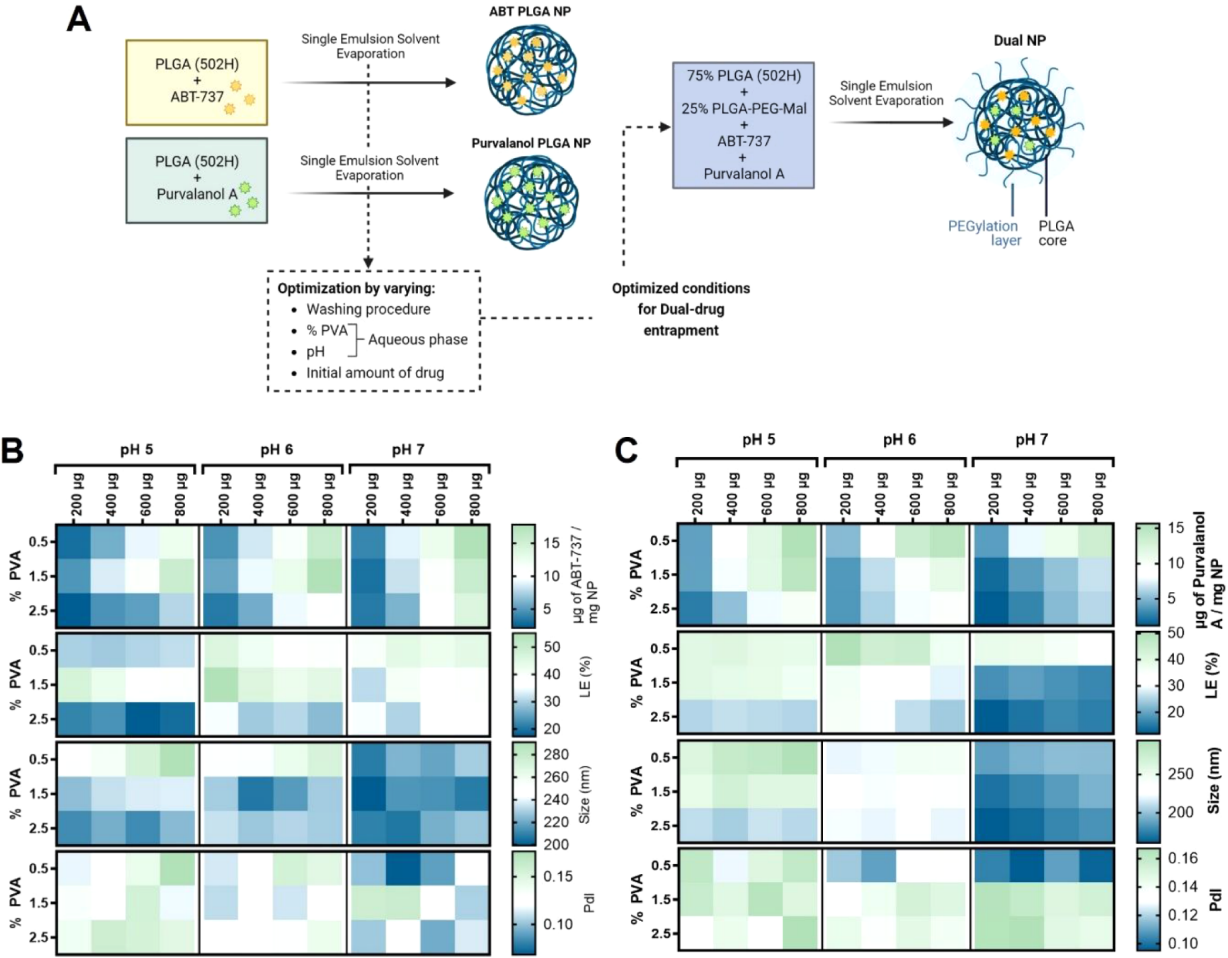
Optimization of single emulsion solvent evaporation method for encapsulation of ABT-737 and Purvalanol A. (A) Schematic overview of the preparation process of ABT PLGA NPs and Purvalanol PLGA NPs as intermediate formulations for optimization of process parameters for the dual-drug entrapment in PEGylated NPs. (B,C) Characterization of ABT PLGA NPs and Purvalanol PLGA NPs, respectively, in terms of the amount of drug entrapped (μg of drug/mg of NP), loading efficiency (LE, %), nanoparticle size (nm), and polydispersity index (PdI). Data are presented as mean values of *n* = 3.

**Figure 3:**
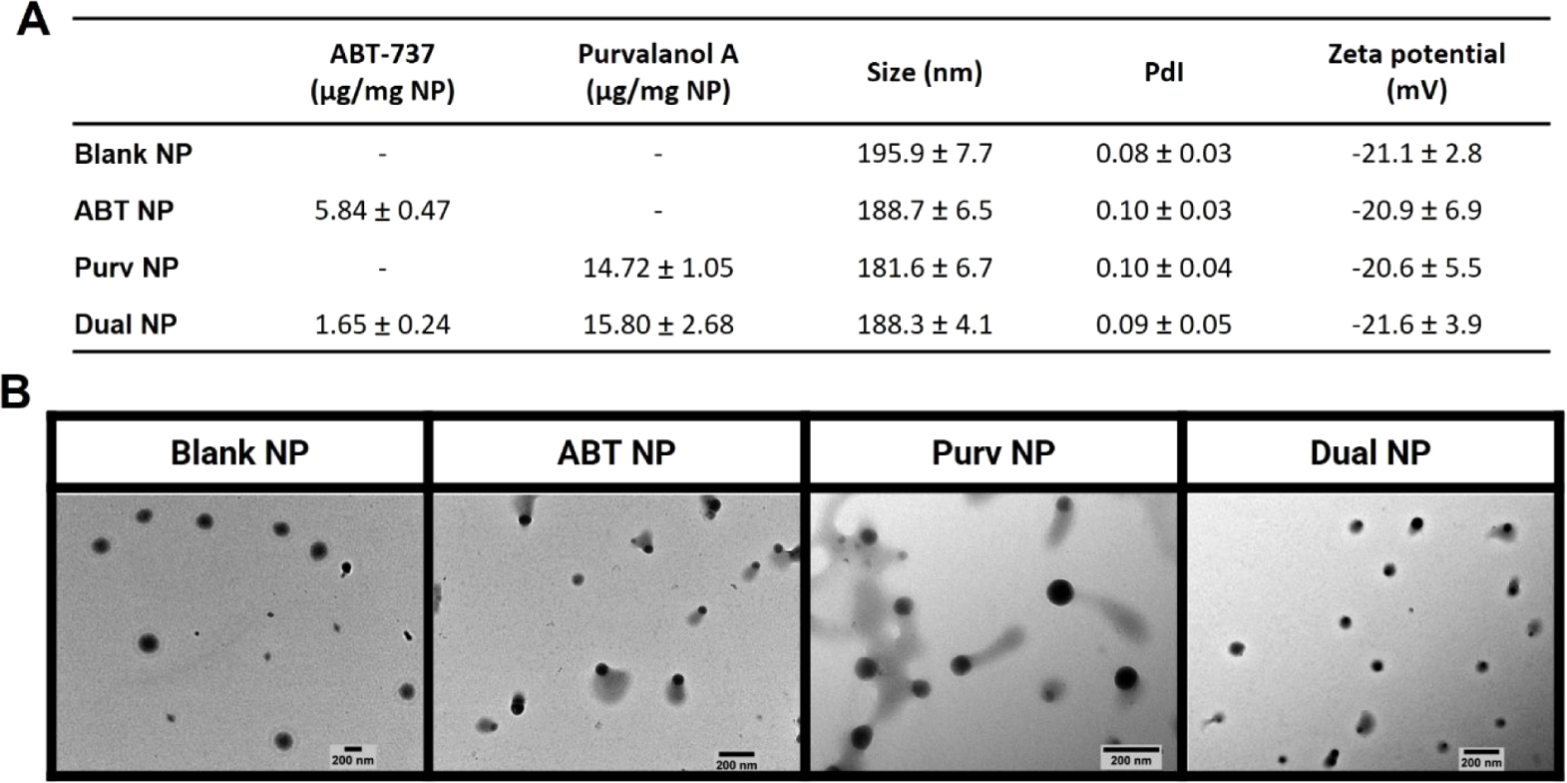
Characterization of PLGA-based PEGylated formulations with and without ABT-737 and/or Purvalanol A loading. (A) Table summarizing the unconjugated NPs characteristics regarding drug loading (μg per mg of NP), DLS-measured hydrodynamic diameter, and polydispersity index (PdI) values, and PALS-measured zeta potential values. Data are presented as mean ± SD, from measurements performed in triplicate and averaged from at least *n* = 3. (B) TEM images of the unconjugated nanoformulations, representative of two independent experiments.

Having observed the synergistic effects obtained with the combination of ABT-737 and Purvalanol A, we next formulated both agents within a PLGA-based nanoparticle drug delivery system. Initially, ABT-737 and Purvalanol A were separately encapsulated into PLGA NPs using a single emulsion solvent evaporation method with varying formulation parameters, to determine the optimal conditions to be employed for the successful coencapsulation of drugs (Figure 2A). While a reduction in the percentage of PVA led to increased encapsulation of both drugs (Figure 2B,C), increasing the pH of the aqueous phase resulted in a general increase in ABT-737 encapsulation (Figure 2B), accompanied by a decrease in Purvalanol A entrapment (Figure 2C). Moreover, increasing the pH overall resulted in the formation of smaller NPs. Previous studies reported that increasing PVA concentration increased the organic solvent/water interfacial tension resulting in smaller NPs, which encapsulated less drug.^23,33^ The aqueous phase pH, on the other hand, is frequently adjusted in an attempt to protonate or deprotonate certain groups to increase entrapment,^23,33^ but it is rarely found to have an impact on nanoparticle size. With the objective of maximizing drug entrapment/loading efficiency and minimizing particle size and polydispersity, 0.5% of PVA in MES buffer at pH 7 was chosen as the optimal aqueous phase for further nanoformulation of both drugs. Having determined the optimal conditions for entrapment of ABT-737 and Purvalanol A within PLGA NPs, the next goal was to develop a dual-drug nanoformulation whereby both drugs were coencapsulated at a synergistic ratio. For this formulation, we also incorporated a PLGA-PEG-maleimide copolymer to facilitate antibody coupling to the NPs in downstream studies and also to introduce a hydrophilic polyethylene glycol (PEG) corona to the PLGA NPs. Despite the successful encapsulation of both drugs within PLGA, the hydrophobic nature of the polymer is known to induce opsonization, promoting NP recognition and clearance by the mononuclear phagocyte system,^34^ while PEG is known to confer stealth properties to NPs by reducing opsonization and therefore enhancing the NPs circulation time.^16,35^ PEGylated nanoformulations were successfully developed encapsulating both drugs (dual NPs) at a molar ratio within the previously tested synergistic range (1:20), as well as nonloaded (blank NP), ABT-737-loaded (ABT NP), and Purvalanol A-loaded NPs (Purv NP), which were used as controls. Overall, the formulations displayed similar sizes, within the desired size range for endocytosis, at around 200 nm,^36^ and polydispersity values, analyzed both via DLS (Figure 3A) and complementary NTA characterization (Figure S3A). Likewise, no significant differences were observed in their zeta potentials (Figure 3A) and morphologies (Figure 3B).

The release profile of the dual NPs was then evaluated at predetermined intervals, up to 48 h, at 37 °C in PBS/10% FBS. Purvalanol A notably exhibited a faster release than ABT-737 within the first hour of the study (Figure S4A). Between 3 and 48 h, however, both drugs demonstrated a slower release with the majority of both having been released by 48 h. Importantly, by the end of the first hour of the study, the molar ratio of drugs released was within the synergistic tested range, and it was then maintained in the lower limit of that range throughout the remaining study time (Figure S4B). Next, the stability of the dual NPs was assessed upon storage of pellets at RT, 4 °C, or −20 °C (Figure S5). For both drugs, no major losses were observed over the course of the study, regardless of the storage condition (Figure S5A,B). In addition, the mean diameter of the NPs remained stable throughout all conditions and time points (Figure S5C), as well as their PdI values, which remained below 0.2 by the end of the study, demonstrating a monodisperse nanoparticle suspension (Figure S5D) and zeta potential, which only showed minor fluctuations (Figure S5E).

### Dual-Loaded NP Treatment Enhances Apoptotic Cell Death

**Figure 4:**
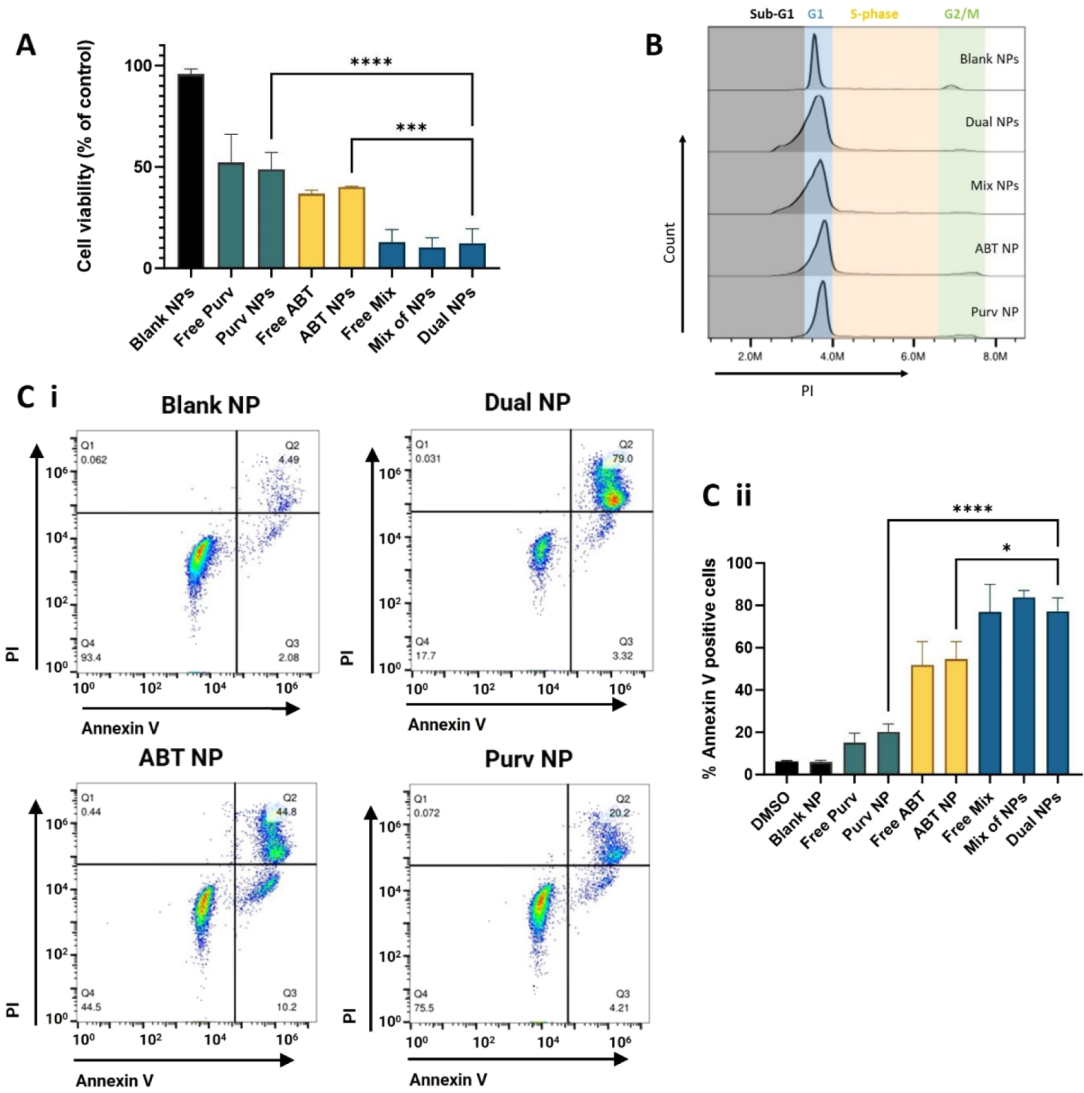
Dual-loaded nanoparticle treatment enhances cell death in pediatric AML cancer models. MV4-11 cells were treated for 72 h with 50 nM of ABT-737, 1.32 μM of Purvalanol A, or their combination, as free drugs or in single-drug loaded NPs, as well as in a dual-drug loaded formulation (dual NP) or as a mixture of the single-drug loaded nanoparticles (mix of NPs). Upon treatment, cells were analyzed for (A) cell viability, by CellTiter-Glo, (B) cell cycle effects via flow cytometry, or (C) Annexin V/PI via flow cytometry: (i) representative dot plots of MV4-11 cells treated with the nanoformulations, (ii) bar chart of percentage of Annexin V-stained cells, considered apoptotic, upon treatment with the nanoformulations and controls. Data are presented as mean ± SD, *n* = 3.

To investigate the effects of the dual-drug loaded NPs on cell death, equivalent amounts of ABT-737 and Purvalanol A, both as free drugs and encapsulated as single- or dual-loaded NPs were added to MV4-11 cells for 72 h treatments. Cell viability analysis confirmed that the drugs were similarly potent whether encapsulated or not, confirming that the entrapped drugs remained active. Importantly, the dual NPs showed significantly increased cell toxicity compared with the single drug-loaded NP formulations (Figure 4A). Further cell cycle analysis highlighted the increased population of cells in the sub-G1 phase when treated with dual NPs, especially when compared to treatment with ABT NPs or Purv NPs, which led only to minimal sub-G1 phase increase or slight cell cycle halt in G1 phase, respectively (Figure 4B). This sub-G1 population growth can indicate an increase in the percentage of apoptotic cells, which was further verified by Annexin V/PI staining assays (Figure 4C). These showed significantly increased apoptotic cell death upon treatment with dual NPs, with the vast majority of cells exhibiting a late-stage apoptotic phenotype, illustrated by double positive staining (Figure 4Ci). Interestingly, we noticed that similar results were obtained following treatment with dual NPs or the mixture of single-loaded NPs at the same drug concentrations (mix of NPs), implying consistent cell uptake of all nanoformulations, as observed by different groups codelivering other entrapped drugs.^37,38^ Similar trends were also observed on the MOLM-13 and MOLM-14 cells (Figures S6 and S7, respectively). Notably, despite reporting no *in vitro* differences in the therapeutic efficacy of dual-drug loaded versus a mix of single-loaded formulations, both Kim et al.^37^ and Zhang et al.^38^ observed significantly greater efficacy of dual-loaded NPs *in vivo*, indicating that only this strategy allowed the codelivery of drugs at a synergistic drug ratio. The success of this codelivery design has also been proven in the clinic, with the approval of CPX-351, which provided synergistic treatment benefits and improved survival over its small molecule combination counterpart in AML.^12^

### Development and Optimization of CD33 NPs with Increased Binding to CD33

**Figure 5:**
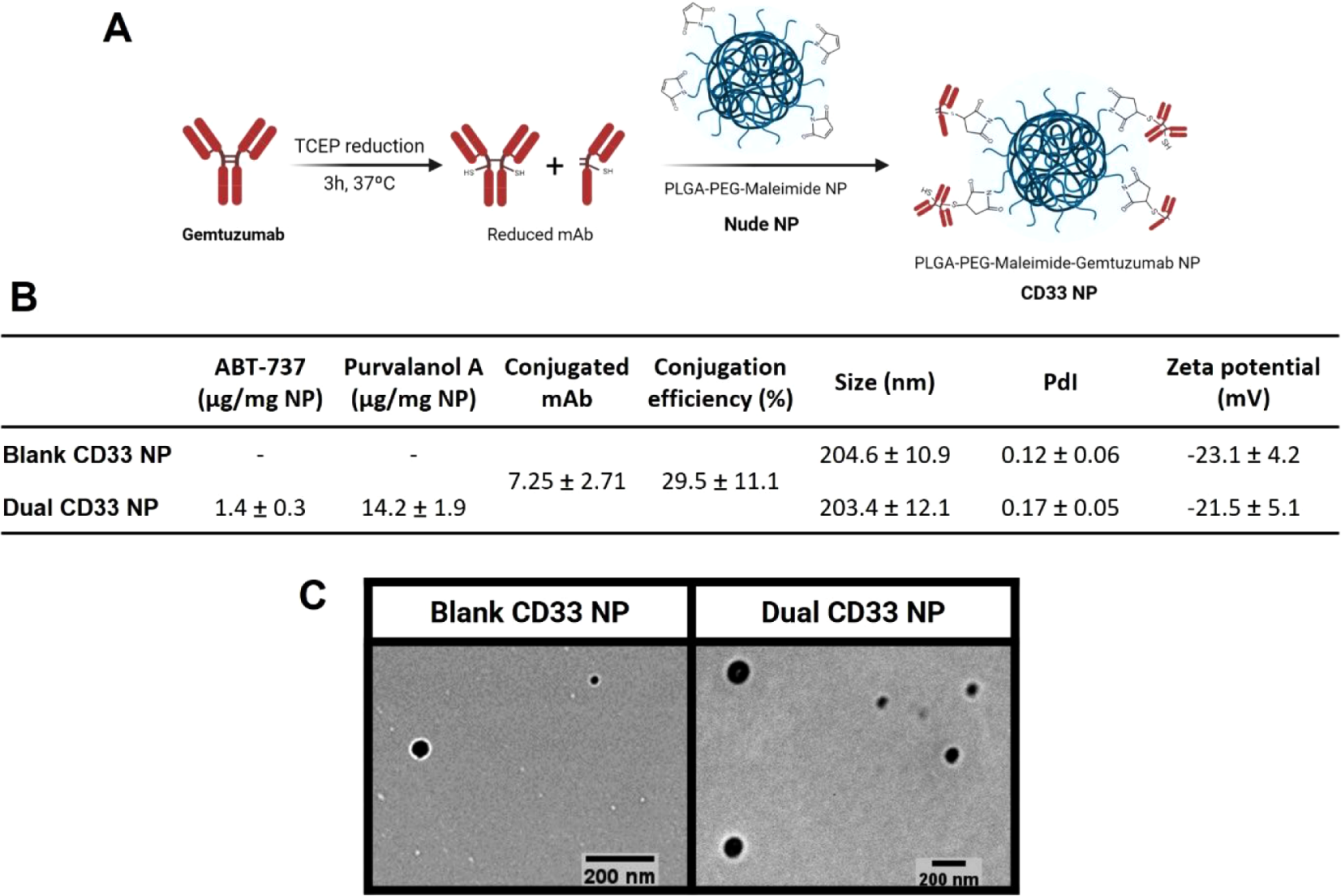
Preparation and characterization of CD33 NPs. (A) Schematic overview of the antibody–nanoparticle conjugation process, using maleimide–thiol chemistry. (B) Table summarizing conjugated NPs characteristics in terms of drug loading, amount of antibody conjugated obtained via Micro BCA, DLS-measured hydrodynamic diameter (nm), and polydispersity index (PdI) values, and PALS-measured zeta potential values. Data are presented as mean ± SD, from measurements performed in triplicate and averaged from at least *n* = 3. (C) TEM images of CD33 NPs, representative of two independent experiments.

To enhance the targeting of the dual NPs to AML cells, we next aimed to conjugate anti-CD33 mAb gemtuzumab to their surface. CD33 is a well-known target for AML that has been widely explored in the development of novel targeted therapies^10,39,40^ and validated in the clinic through the use of GO.^41^ The PLGA-PEG-maleimide copolymer incorporated within the dual NP formulation granted an opportunity for antibody conjugation via reaction with free thiol groups obtained by reduction of the antibody interchain disulfide bridges^42^ (Figure 5A). This cysteine-based method of conjugation resulted in gemtuzumab-conjugated NPs (CD33 NPs) with the ability to bind to CD33-Fc and with physicochemical properties within the desired ranges (Figure S8). Binding of the nanoformulations to recombinant CD33-Fc was evaluated using the fluorescent properties of rhodamine 6G-loaded NPs as readouts of binding of NPs to immobilized CD33 in FLISA assays. Using this method, the binding of cysteine-conjugated NPs was superior to that of CD33 NPs formulated using a traditional lysine-NHS conjugation approach (Figure S8).

Using the previously employed 25% (w/w) of PLGA-PEG-maleimide copolymer conjugated with 25 μg of antibody per mg of NP, an optimal balance was achieved between maximizing NP binding and conjugation efficiency while minimizing size and polydispersity (Figure S9), resulting in blank and dual drug-loaded CD33 NPs with approximately 30% antibody conjugation efficiency (Figure 5B). SDS-PAGE analysis further confirmed the bioconjugation and revealed the presence of mainly half antibody fragments on the surface of the NPs, as suggested by the detection of a 75 kDa band on the conjugated NPs (Figure S10). This led to a diameter increase of around 10–15 nm compared with the previously developed nude formulations (Figures 3A and 5B). However, CD33 NPs still showed an acceptable size of around 200 nm and were monodisperse, as observed both via DLS (Figure 5B) and NTA (Figure S3B). Conjugation also resulted in no significant alteration in the zeta potential or morphology of the NPs (Figure 5B,C). Importantly, dual CD33 NPs retained a synergistic drug entrapment, corresponding to a molar ratio of 1:21, at a sufficient concentration to exert complete cell death using low amounts of NPs, at which negligible toxicity is elicited by the polymer (Figure S11).

### Binding Evaluation of Optimized CD33 NP Formulation to Recombinant CD33-Fc

**Figure 6:**
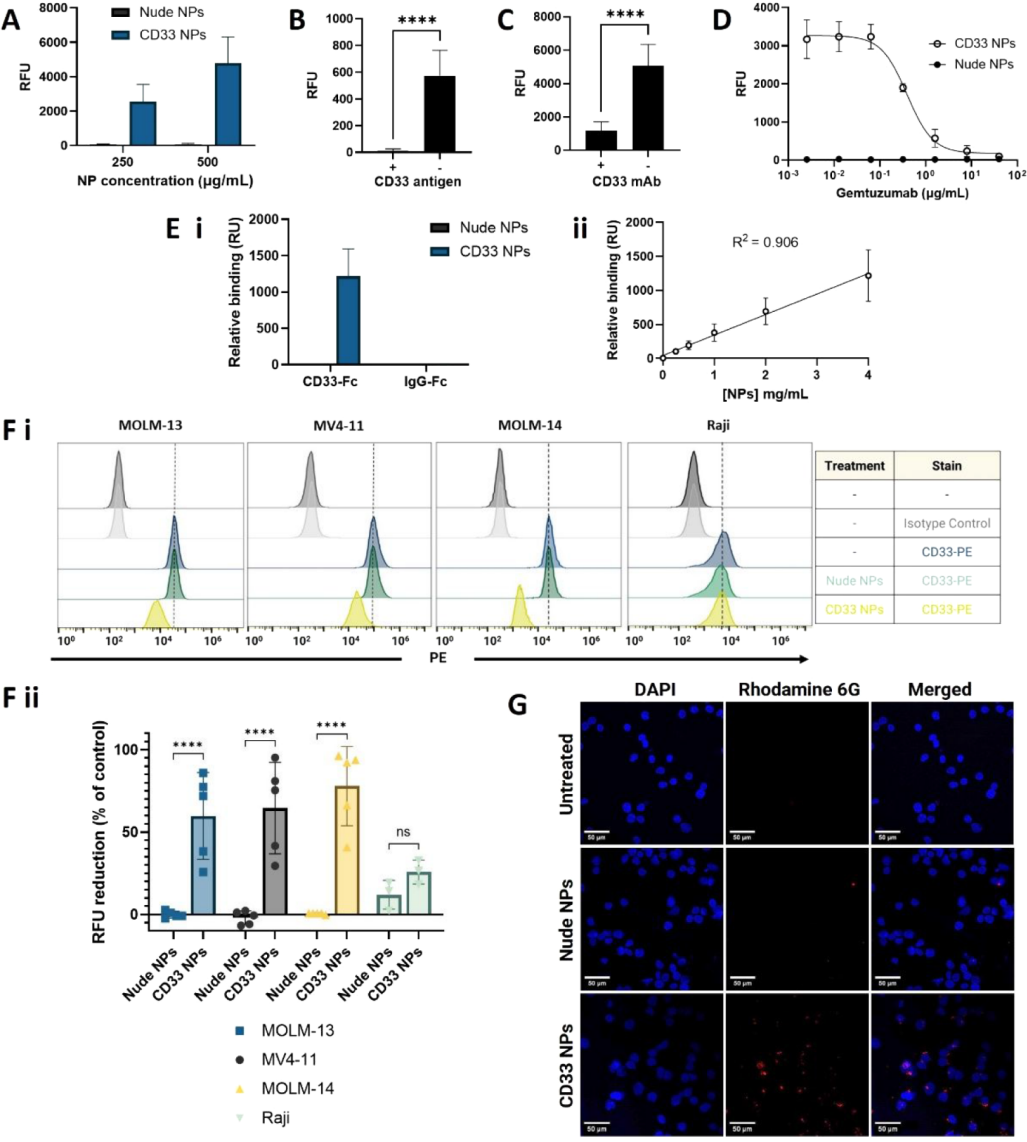
Binding of nanoformulations to recombinant CD33-Fc and CD33-expressing cells. (A–D) Binding of rhodamine 6G-loaded NPs to recombinant CD33-Fc in FLISA assays: (A) dose-dependent binding, (B) binding of NPs (50 μg polymer/mL) ± preincubation with CD33-Fc (10 μg/mL), (C) binding of NPs (500 μg polymer/mL) ± preblock with CD33 mAb (40 μg/mL), (D) binding of NPs (500 μg polymer/mL) in competition with varying concentrations of gemtuzumab (0.00256–40 μg/mL). Data are presented as mean ± SD, *n* = 3. (E) Binding of nonfluorescent nanoformulations to CD33-Fc and IgG-Fc evaluated by SPR: (i) binding of the NPs at 4 mg/mL to CD33-Fc and IgG-Fc, (ii) binding of CD33 NPs to CD33-Fc at varying concentrations, with linear regression and corresponding goodness of fit (*R*^2^). Binding is presented as response relative to baseline observed 5 s before the end of the injection period. Data are presented as mean ± SD, *n* = 2. (F) Cells were treated with blank CD33 or nude NPs (750 μg polymer/mL) for 1 h at 4 °C. Then, cells were washed, stained with PE-labeled anti-CD33 antibody or isotype control antibody, and PE-fluorescence was analyzed by flow cytometry. Representative histograms are shown for each condition tested, (i), as well as the corresponding reduction of fluorescence compared with the positive stained control observed after treatment with the NPs for each cell line (ii). (G) Confocal microscopy images of MOLM-13 cells treated with 500 μg polymer/mL rhodamine 6G-loaded NPs for 1 h at 4 °C, followed by a washing step and a further 2 h-incubation at 37 °C. Scale bar is 50 μm, and blue and red staining denote cell nuclei and nanoparticles, respectively. Representative data from *n* = 2.

Having developed and characterized CD33 NPs, an additional set of FLISA assays was employed to evaluate the binding ability and specificity of the formulation. Upon incubation of rhodamine 6G-loaded (as a surrogate to loaded drugs) fluorescent NPs with CD33-Fc immobilized on microtiter plate wells, we observed a dose-dependent binding of CD33 NPs, while nude NPs showed negligible binding (Figure 6A). Next, competition assays were performed on the conjugated NPs, where free CD33-Fc antigen was added to the NPs prior to being added to the CD33-Fc-coated plates (Figure 6B), or the plate was preblocked with free gemtuzumab prior to the addition of the NPs (Figure 6C). In both cases, these preincubations resulted in a significant decrease in NP binding. Finally, varying concentrations of free gemtuzumab were mixed with conjugated NPs and simultaneously added to the plate, which resulted in a progressive inhibition of binding with the increasing concentration of free antibody (Figure 6D). SPR was then used for a complementary analysis of the nanoparticle binding to CD33-Fc immobilized on a carboxymethylated dextran chip, and binding to IgG-Fc was also examined as a negative control. These studies corroborated the results observed via FLISA, demonstrating the ability of CD33 NPs to bind specifically to CD33-Fc, and not to the negative control, as well as the negligible binding of nude NPs (Figure 6Ei). Moreover, we observed a linear correlation (*R*^2^ = 0.906) between the NP concentration and CD33 binding (Figure 6Eii). Taken together, these results confirm the targeting ability of the developed CD33 NPs through specific engagement with CD33-Fc.

### Nanoparticle Binding and Internalization in CD33-Expressing AML Cells

Cell-based assays were then employed to evaluate the binding ability of the nanoformulations in a physiological setting. The human AML cell lines MV4-11, MOLM-13, and MOLM-14 employed previously were also suitable models for these studies due to their varying surface expression levels of CD33 (Figure S12). Raji cells were also used as a negative control due to their very limited CD33 surface expression (Figure S12). Unloaded nude or CD33 conjugated NPs were incubated with cells for 1 h at 4 °C, to prevent endocytosis, prior to staining with a PE-labeled anti-CD33 antibody to visualize the surface CD33 receptor remaining unoccupied by the NPs (Figure 6F). After treatment with nude NPs, CD33 antibody binding was detected as in the untreated cells. On the other hand, after incubation with CD33 conjugated NPs, there was a marked reduction in the CD33 staining, corresponding to approximately 60%, 65%, and 78% reduction in fluorescence for MOLM-13, MV4-11, and MOLM-14 cells, respectively (Figure 6Fii). Conversely, for the lower CD33 expressing Raji cells, the CD33 NP treatment did not lead to a significant reduction in the level of CD33 staining. Taken together, these data suggested that CD33 NPs could successfully bind to CD33 receptors on the surface of the cells, impeding the binding of the PE-tagged antibody.

In order to demonstrate not only binding but also internalization of the developed nanoformulations, further studies were employed using confocal microscopy on MOLM-13 cells, which were chosen due to their higher surface expression of CD33. When preincubated with the cells at 4 °C, followed by a further incubation at 37 °C for 2 h to enable internalization, there was a clear association of fluorescent rhodamine 6G-loaded CD33 NPs with cells, which was not evident with nude NPs (Figure 6G). Furthermore, z-stack imaging confirmed the colocalization of CD33 NPs in the cell nuclei, indicating that targeted NPs were successfully internalized, and not just adhered to the cell surface (Figure S13). In an alternative approach, cells were treated with nanoparticles for increasing periods of time at 37 °C. After 30 min of incubation, there were no major differences observed between nude and CD33 NPs (Figure S14). However, a gradual time-dependent increase in the number and intensity of fluorescent points was observed in the cells treated with CD33 NPs, demonstrating faster and greater internalization of the nanoparticles upon gemtuzumab conjugation (Figure S14).

Overall, these results demonstrate how gemtuzumab conjugation improved receptor-mediated recognition and internalization of the nanoparticles. Similarly, Koyakutty’s group demonstrated receptor-mediated uptake of their CD33-targeted NP through antibody-blocking experiments, as well as targeted cytotoxicity to CD33-positive cells only.^43^ In a different approach, an oridonin-conjugated anti-CD33 antibody presented selective uptake in MOLM-13 and MV4-11 cells but not in a CD33-negative cell line.^44^

### Targeted Delivery of ABT-737 and Purvalanol A to CD33-Expressing Pediatric AML Cells

**Figure 7:**
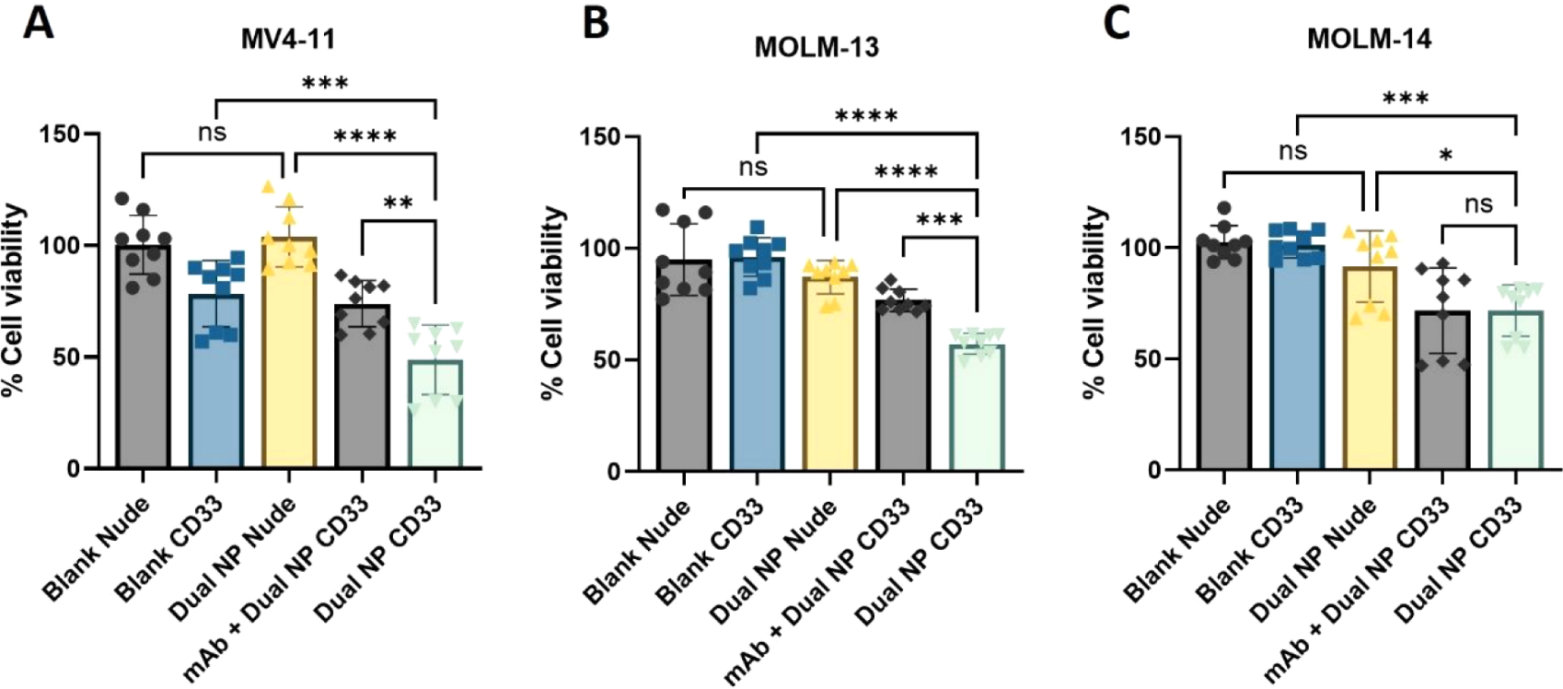
Targeted delivery of synergistic drug combination within dual CD33 NPs. (A) MV4-11, (B) MOLM-13, and (C) MOLM-14 cells were treated with blank or dual-loaded conjugated (CD33 NP) or nonconjugated (nude NP) nanoparticles for 1 h at 4 °C. Then, cells were washed, counted, and reseeded for 72 h of incubation at 37 °C, prior to measurement of cell viability. Where appropriate, cells were preincubated with 5 μg of free-gemtuzumab for 15 min at 4 °C and washed, prior to NP incubation. Data are presented as mean ± SD, *n* = 3.

In a final series of studies, the ability of the dual CD33 NPs to deliver the coencapsulated drugs in a targeted manner was evaluated. While treatment with dual nude NPs showed no significant cytotoxicity, upon treatment with dual CD33 NPs cell viability was markedly reduced compared to controls including dual nude NPs or blank CD33 NPs in all cell lines tested, indicating that the drugs were delivered in a targeted manner (Figure 7). Furthermore, to confirm that the observed reduction in cell viability was mediated by CD33 targeting, cells were also preincubated with free gemtuzumab prior to treatment with dual CD33 NPs, which partially restored cell viability in MV4-11 and MOLM-13 cells (Figure 7A,B), highlighting the CD33-dependent uptake of the formulation. In MOLM-14 cells; however, this effect was not statistically significant (Figure 7C), perhaps due to their lower CD33 expression and lower sensitivity to the drugs. Other groups have reported the development of CD33-targeted PLGA-based^43^ or lipid-based^45,46^ nanosystems for delivery of different compounds to AML cells. Consistent with our findings, in these studies, CD33-targeting enhanced compound delivery and associated toxicity in the targeted cells.

## Conclusions

In summary, we have validated the synergistic cytotoxicity of ABT-737 and Purvalanol A in pediatric AML cell models and successfully developed a novel polymeric system for their codelivery. This coencapsulation and sustained ratiometric drug release has the potential to add great value to the formulation of ABT-737 and Purvalanol A, not only by overcoming the poor solubility of the compounds, which has contributed to their absence from the clinic so far, but also by delivering the drugs simultaneously, circumventing their potentially dissimilar pharmacokinetic profiles. Indeed, clinically evaluated analogues ABT-263 and roscovitine have reported clearly distinct half-lives of 17 h and 2–5 h, respectively.^18,47^ Additionally, we have shown that the developed formulation can effectively induce apoptotic cell death, and surface conjugation to gemtuzumab resulted in CD33-mediated cell uptake, offering a targeted dual drug-delivery approach. Future work is warranted to evaluate the safety and efficacy of the developed formulation *in vivo*, as well as to gain a better understanding of its pharmacokinetic profile and biodistribution. Besides, determination of the plasma concentrations of the two drugs following nanoformulation administration *in vivo* could help ascertain whether the synergistic ratiometric release observed *in vitro* translates to a more physiologically relevant setting. However, considering the promising therapeutic potential of polymer-based nanotherapeutics such as the docetaxel-loaded PLGA-PEG nanoformulation (BIND-014),^48,49^ the clinical success of the dual-drug delivery design illustrated by CPX-351,^12^ and the enormous progress recently observed in the ADC field supported by several FDA approvals,^50^ targeted drug-delivery therapies such as the one proposed in this work represent an exciting new approach for the treatment of cancer.

## Supporting Information Available

Analytical method developed for drug entrapment quantification, cytotoxic effects of free-drugs in AML cell lines, additional NTA characterization of nanoformulations, drug release profile of ABT-737 and Purvalanol A, NPs stability evaluation, assessment of dual NPs effect on cell death of additional cell lines, characteristics of CD33 NPs obtained using different conjugation chemistries (maleimide vs NHS), optimization of gemtuzumab conjugation to maleimide NPs, targeted NPs characterization with SDS-PAGE, CD33 expression levels across the cell lines used, and additional confocal microscopy NP internalization results (PDF)
