# Supplementary material for: Development of CD33-Targeted Dual Drug-Loaded Nanoparticles for the Treatment of Pediatric Acute Myeloid Leukemia
Source: Biomacromolecules. 2024 Sep 5;25(10):6503–14. doi: 10.1021/acs.biomac.4c00672 (PMC11480974; doi:10.1021/acs.biomac.4c00672)
Supplement: Supplementary file 1 — bm4c00672_si_001.pdf [file bm4c00672_si_001.pdf]

Supporting Information to

Development of CD33-targeted dual-drug loaded nanoparticles for the treatment of paediatric acute myeloid leukaemia

*Ana M. Carvalho[a], Michelle K. Greene[a], Peter Smyth[a], Alexander Mutch[a], Kirsty M. McLaughlin[a], Lauren V. Cairns[a], Ken I. Mills[a], Karen D. McCloskey[a], Christopher J. Scott[a]**

[a] The Patrick G Johnston Centre for Cancer Research, School of Medicine, Dentistry and Biomedical Sciences, Queen's University Belfast, Belfast BT9 7AE, United Kingdom.

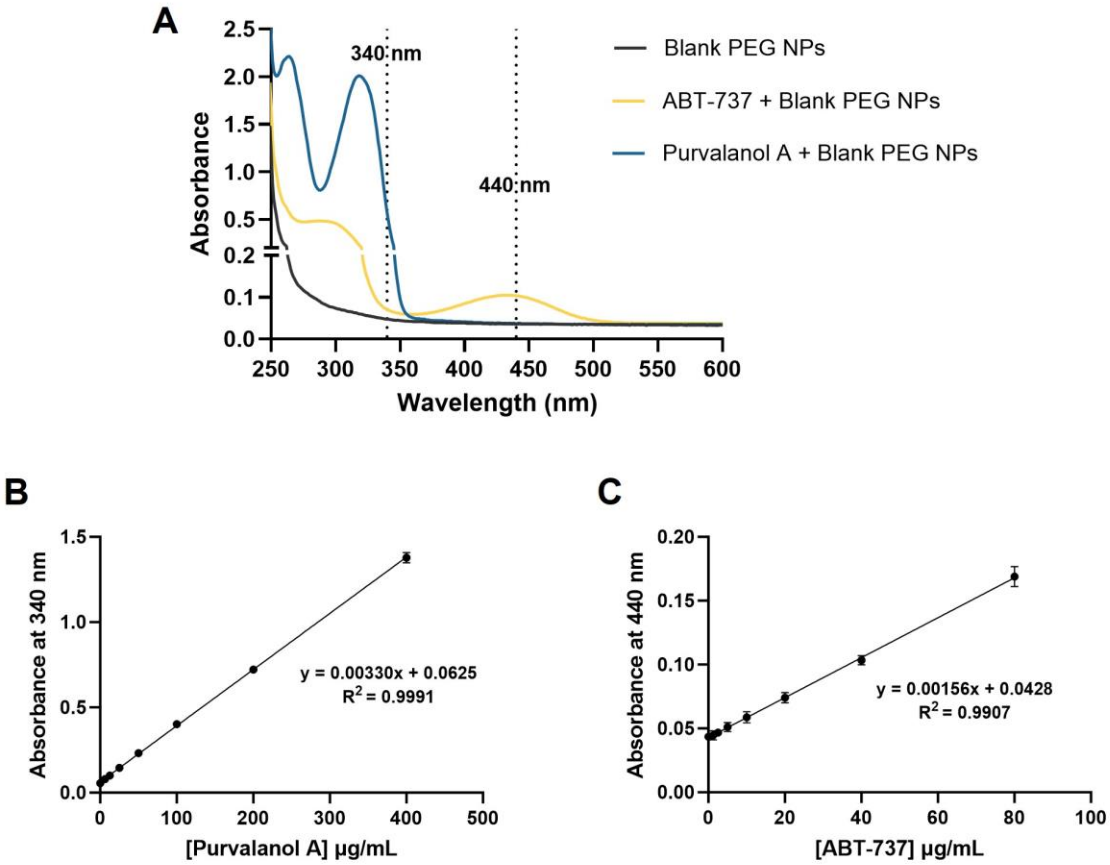

Figure S1. Development of an analytical method for simultaneous quantification of ABT-737 and Purvalanol A entrapment. A) UV-Vis absorption spectra of each drug spiked into ACN:DMSO dissolved Blank PEG NPs and wavelengths selected for measurement of each drug. **B)** and **C)** Standard curves of Purvalanol A and ABT-737, respectively, prepared using varying concentrations of the drugs mixed with dissolved Blank PEG NPs and read at the pre-determined wavelengths. Data is presented as mean ± SD, obtained from duplicates and n=6.

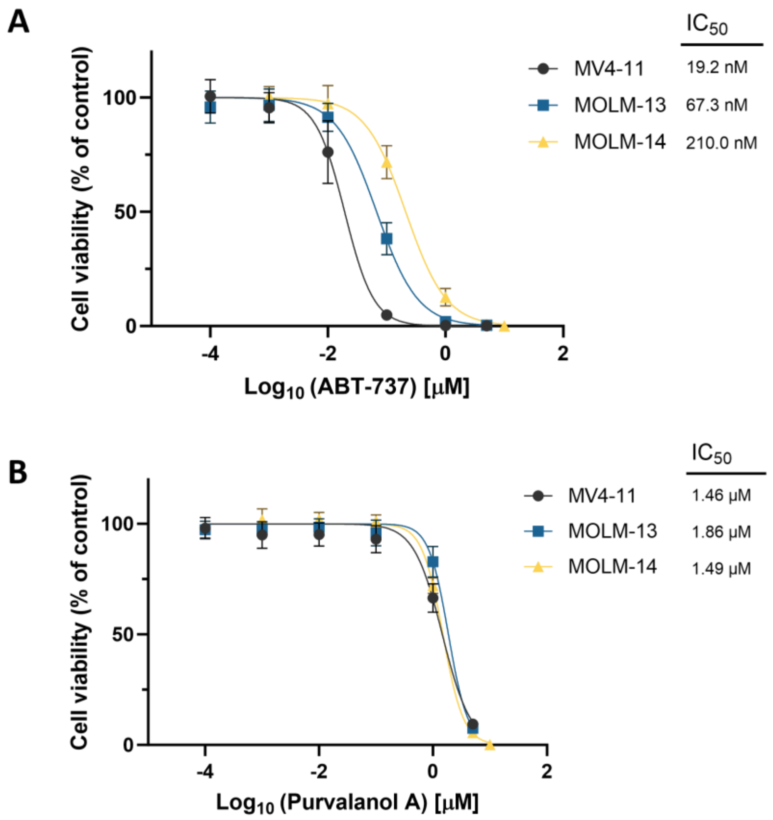

Figure S2. Dose response curves of free drugs in a panel of AML cell lines. MV4-11, MOLM-13 and MOLM-14 cells were treated with **A)** ABT-737 or **B)** Purvalanol A for 72 h, prior to analysis of cell viability using CellTiter-Glo®. Data is presented as mean ± SD, obtained from triplicates and n=3.

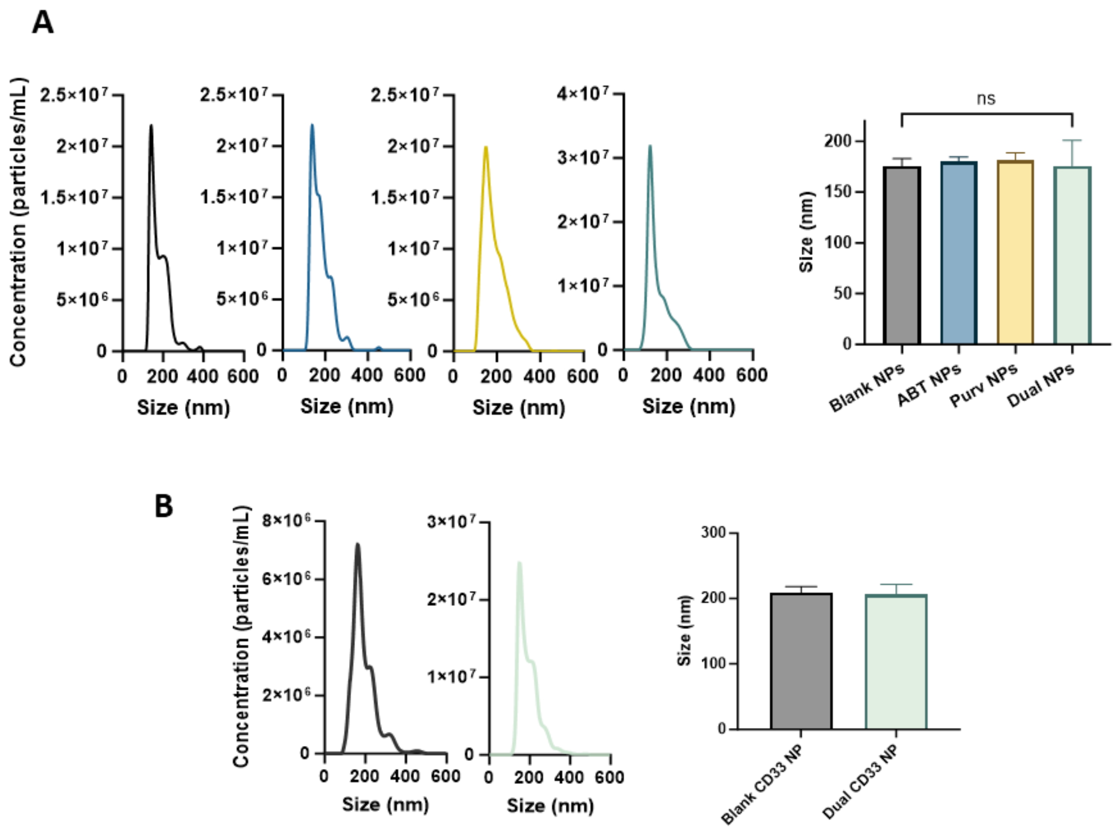

Figure S3. NTA characterization of PLGA-based PEGylated formulations. Characteristics of **A)** unconjugated NPs and **B)** gemtuzumab-conjugated "CD33" NPs. NTA histograms and bar chart presenting the mean diameters ± SE, representative of n=2.

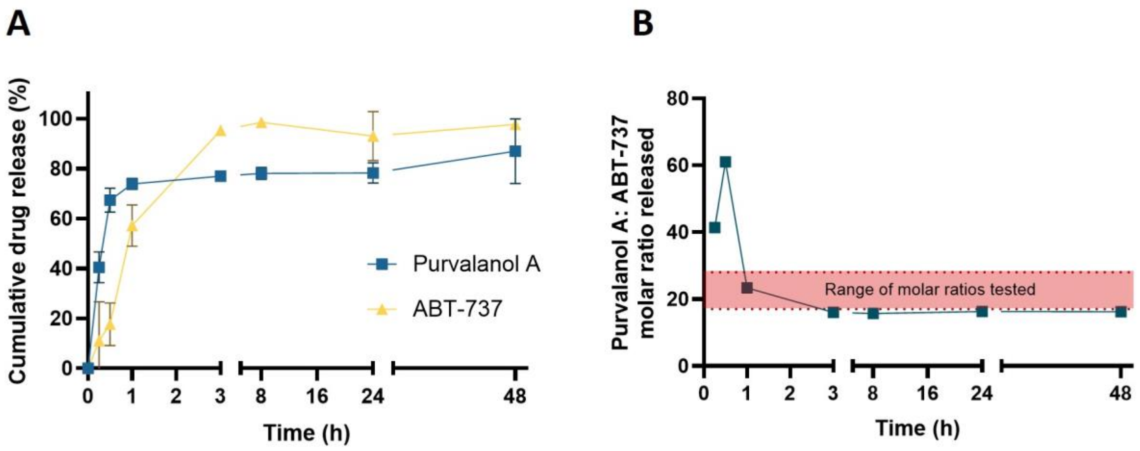

Figure S4. Drug release profile of ABT-737 and Purvalanol A from Dual drug-loaded PLGA-PEG NPs. A) Cumulative release percentage. Dual NPs were resuspended at 1 mg polymer/mL in PBS/10% FBS and incubated at 37ºC under constant agitation at 200 rpm, in Eppendorf vials containing 1.5 mL of NP suspension over the course of 48 h. At each predetermined timepoint, an Eppendorf was collected, centrifuged and the NP pellet washed once with SDS and twice with PBS, prior to drug-loading quantification and release calculation with respect to the initial timepoint. Data is presented as mean ± SD, representative of n=2. **B)** Molar ratio of drugs released from the NPs at each timepoint, calculated from the data obtained in A).

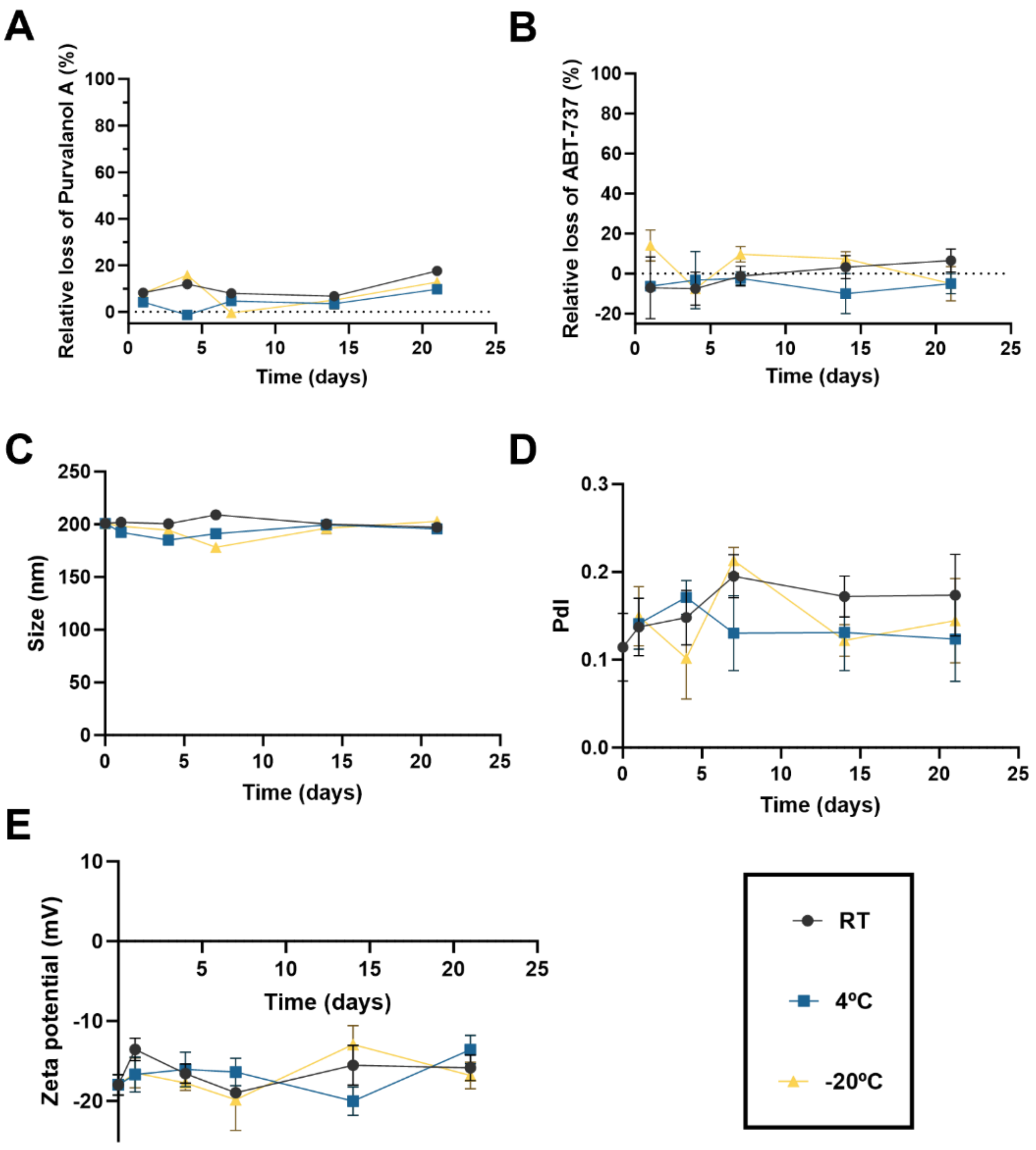

Figure S5. Assessment of Dual NPs stability upon storage at different temperatures. Dual NP pellets were stored at room temperature (RT), 4 ºC, or -20 ºC for up to 21 days and evaluated at predetermined timepoints: **A)** and **B)** percentage loss of Purvalanol A and ABT-737, respectively, relative to the amount of drug quantified in the NPs at day 0; **C)** and **D)** nanoparticle's diameter and polydispersity index (PdI), respectively, evaluated by DLS measurements; **E)** zeta-potential, determined via PALS measurement. Data is presented as mean ± SD, obtained from triplicates and representative of n=2.

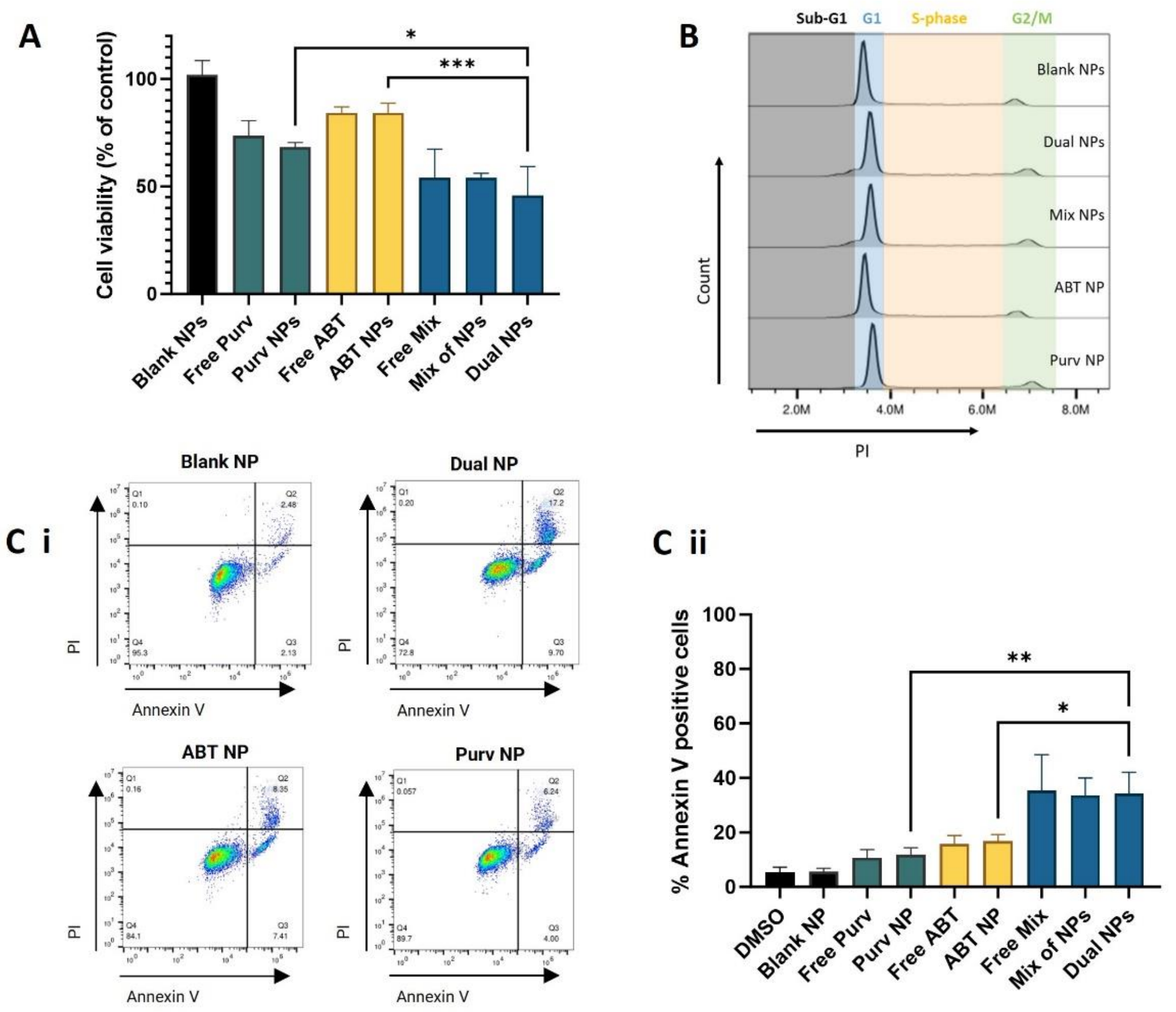

Figure S6. Dual-loaded nanoparticle treatment enhances cell death in MOLM-13 cells. Cells were treated for 72 h with 50 nM of ABT-737, 1.32 uM of Purvalanol A, or their combination, as free drugs or in single-drug loaded NPs, as well as in a dual-drug loaded formulation (Dual NP) or as a mixture of the single-drug loaded nanoparticles (Mix of NPs). Upon treatment, cells were analyzed for **A)** cell viability, by CellTiter-Glo, **B)** cell cycle effects via flow cytometry, or **C)** annexin V/PI via flow cytometry: **i)** representative dot plots of cells treated with the nanoformulations, **ii)** bar chart of percentage of annexin V-stained cells, considered apoptotic, upon treatment with the nanoformulations and controls. Data is presented as mean ± SD, n=3.

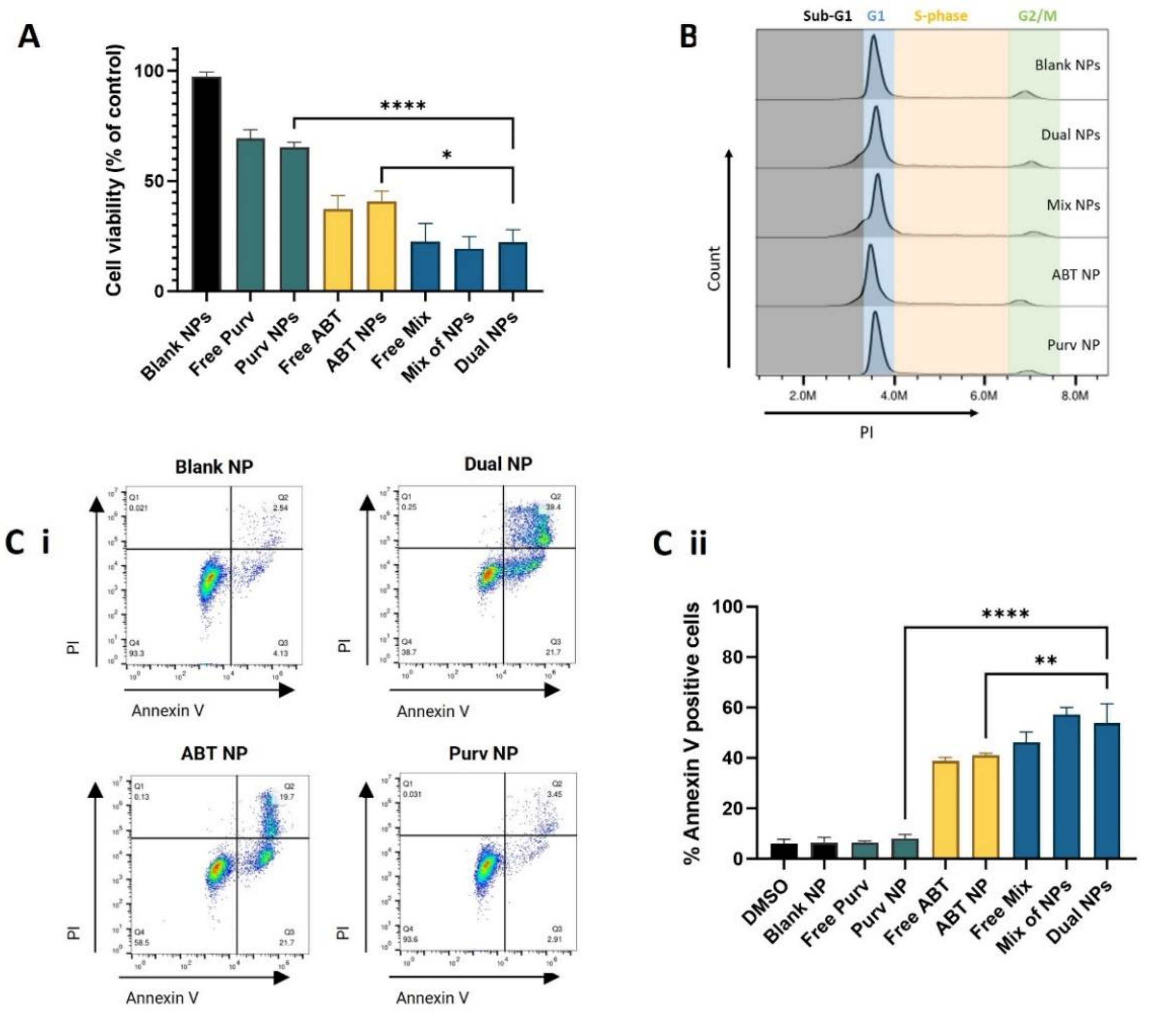

Figure S7. Dual-loaded nanoparticle treatment enhances cell death in MOLM-14 cells. Cells were treated for 72h with 50 nM of ABT-737, 1.32 uM of Purvalanol A, or their combination, as free drugs or in single-drug loaded NPs, as well as in a dual-drug loaded formulation (Dual NP) or as a mixture of the single-drug loaded nanoparticles (Mix of NPs). Upon treatment, cells were analyzed for **A)** cell viability, by CellTiter-Glo, **B)** cell cycle effects via flow cytometry, or **C)** annexin V/PI via flow cytometry: **i)** representative dot plots of cells treated with the nanoformulations, **ii)** bar chart of percentage of annexin V-stained cells, considered apoptotic, upon treatment with the nanoformulations and controls. Data is presented as mean ± SD, n=3.

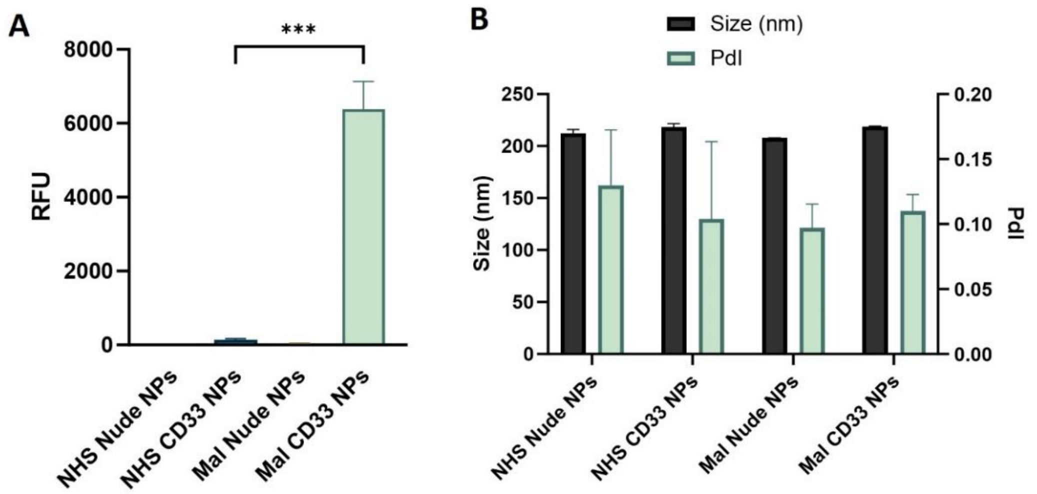

Figure S8. Characteristics of antibody-nanoparticle conjugates obtained via N-hydroxysuccinimide (NHS) or maleimide (Mal) based conjugation chemistries. A) Binding of fluorescent NPs (500 µg polymer/mL) to immobilized CD33 antigen via FLISA. **B)** Sizes and PdI values of unconjugated (Nude) and conjugated (CD33) NPs. Data is presented as mean ± SD, n=3.

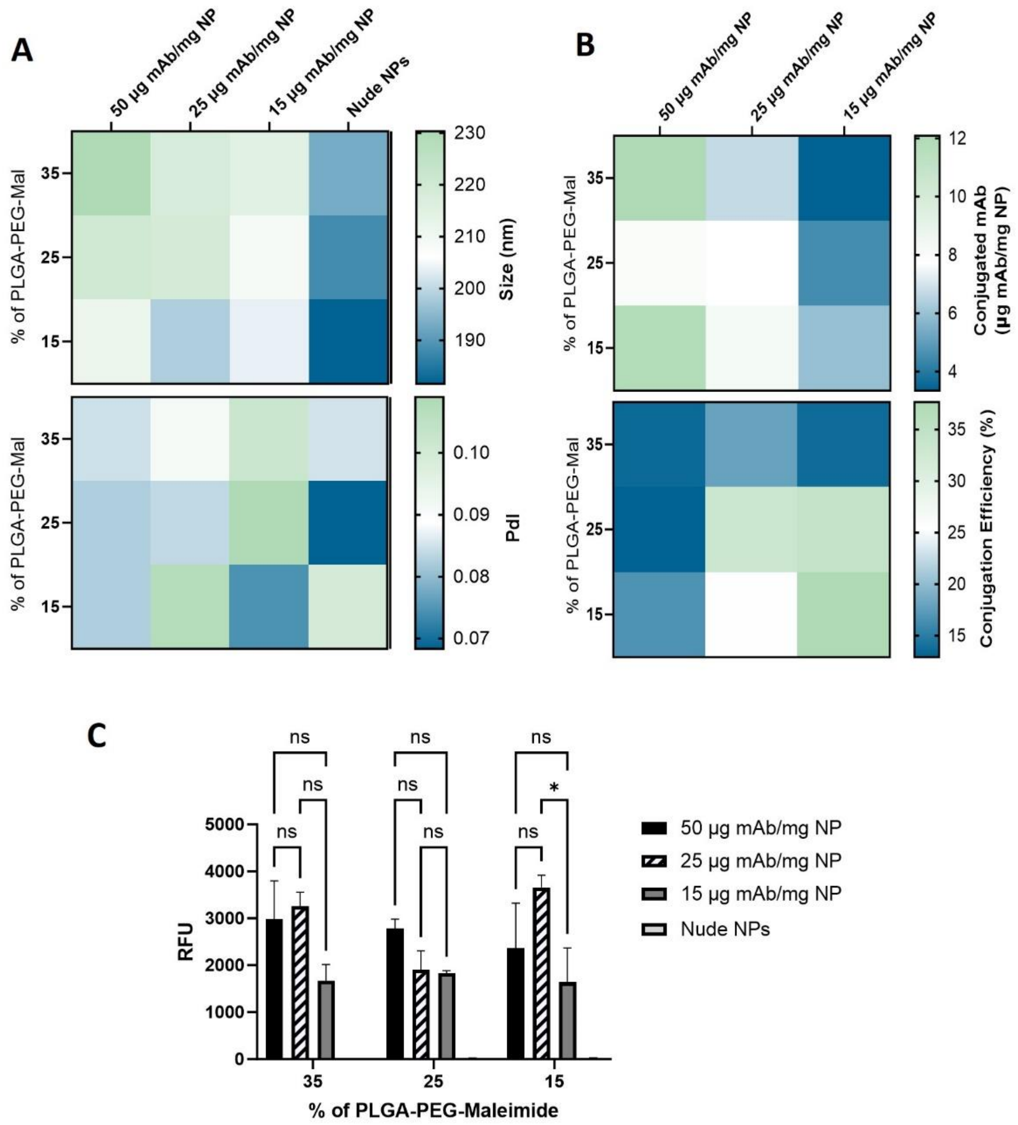

Figure S9. Effects of varying percentage of PLGA-PEG-maleimide and initial amounts of gemtuzumab added to NPs for conjugation. A) DLS-measured NPs mean diameter (size) and PdI values. **B)** Amount of Gemtuzumab conjugated (µg per mg of NPs) and corresponding conjugation efficiency (%) obtained via Micro BCA. **C)** Binding of fluorescent Rhodamine 6G-loaded NPs (250 µg polymer/mL) to immobilized CD33 antigen via FLISA. Data is presented as mean ± SEM, n=3.

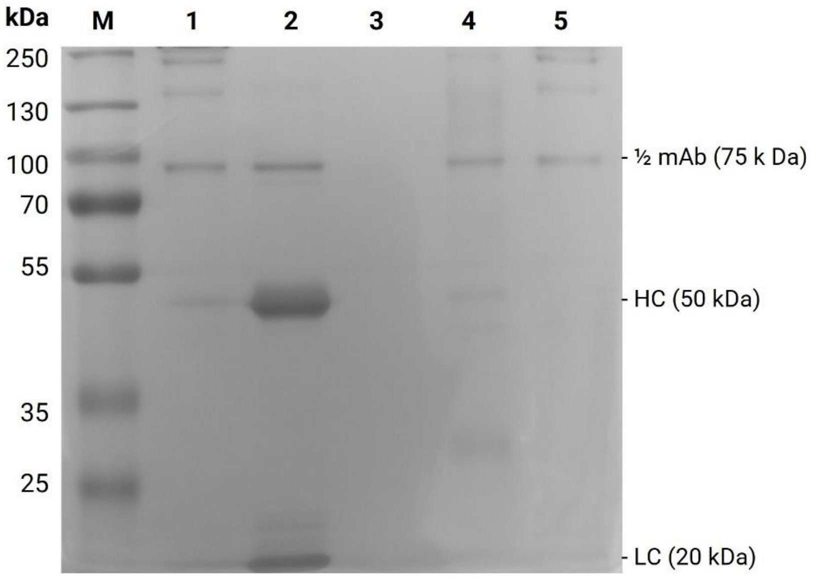

Figure S10. Non-reducing SDS-PAGE of antibody pre-treatment and NP-conjugation. M: Molecular weight marker; 1: Native gemtuzumab; 2: TCEP pre-treated gemtuzumab; 3: Blank Nude NPs; 4: Blank CD33 NPs; 5: Blank Nude NPs + Native gemtuzumab.

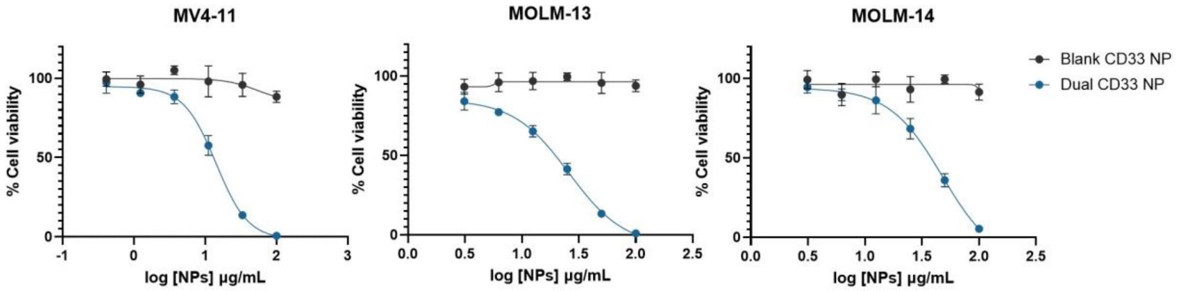

IC_{50} (μg NP/mL)	MV4-11	MOLM-13	MOLM-14
**Dual CD33 NP**	14.15	25.88	47.68

Figure S11. Cytotoxic effects of Dual CD33 NPs and Blank CD33 NPs in AML cells. A) Dose response curves of MV4-11, MOLM-13, and MOLM-14 cells after 72-hour treatments with dual-loaded targeted NPs, as well as Blank targeted NPs. Cell viability was measured via CellTiter-Glo and presented as relative to untreated control cells. **B)** Table summarising IC_{50} values obtained for each cell line. Data is presented as mean ± SD, obtained from triplicates and n=3.

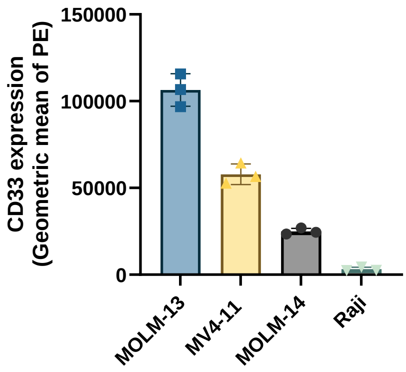

Figure S12. Varying expression levels of CD33 across a panel of cells lines. Cells were stained with anti-CD33 PE-labelled antibody and analysed by flow cytometry. Data is presented as mean ± SD, n=3.

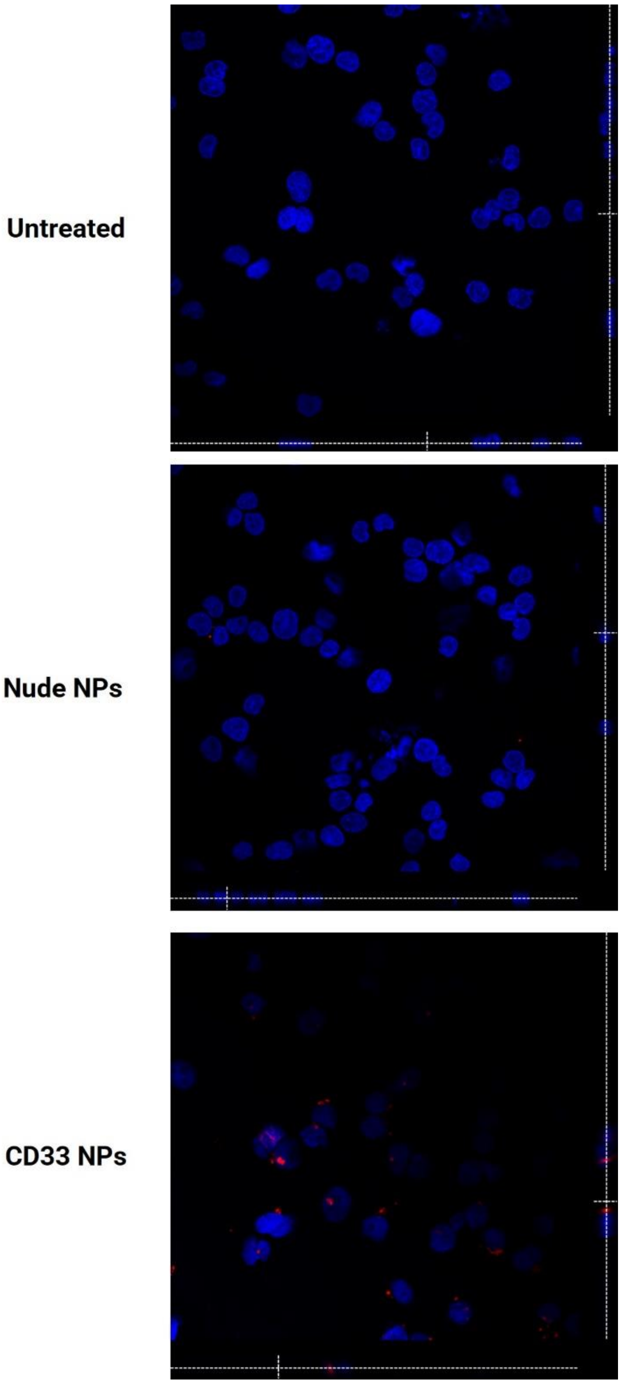

Figure S13. Receptor mediated internalization of rhodamine 6G-loaded nanoparticles in MOLM-13 cells via confocal microscopy. Cells were treated with 500 µg polymer/mL of Nude or CD33-targeted rhodamine 6G-loaded NPs for 1 h at 4ºC, followed by a washing step and a further 2 hour-incubation at 37ºC. Blue and red staining denote cell nuclei and nanoparticles, respectively. Cross-sectional images of the cells were taken through the z-plane using Z-stack imaging. Representative data from 2 independent experiments.

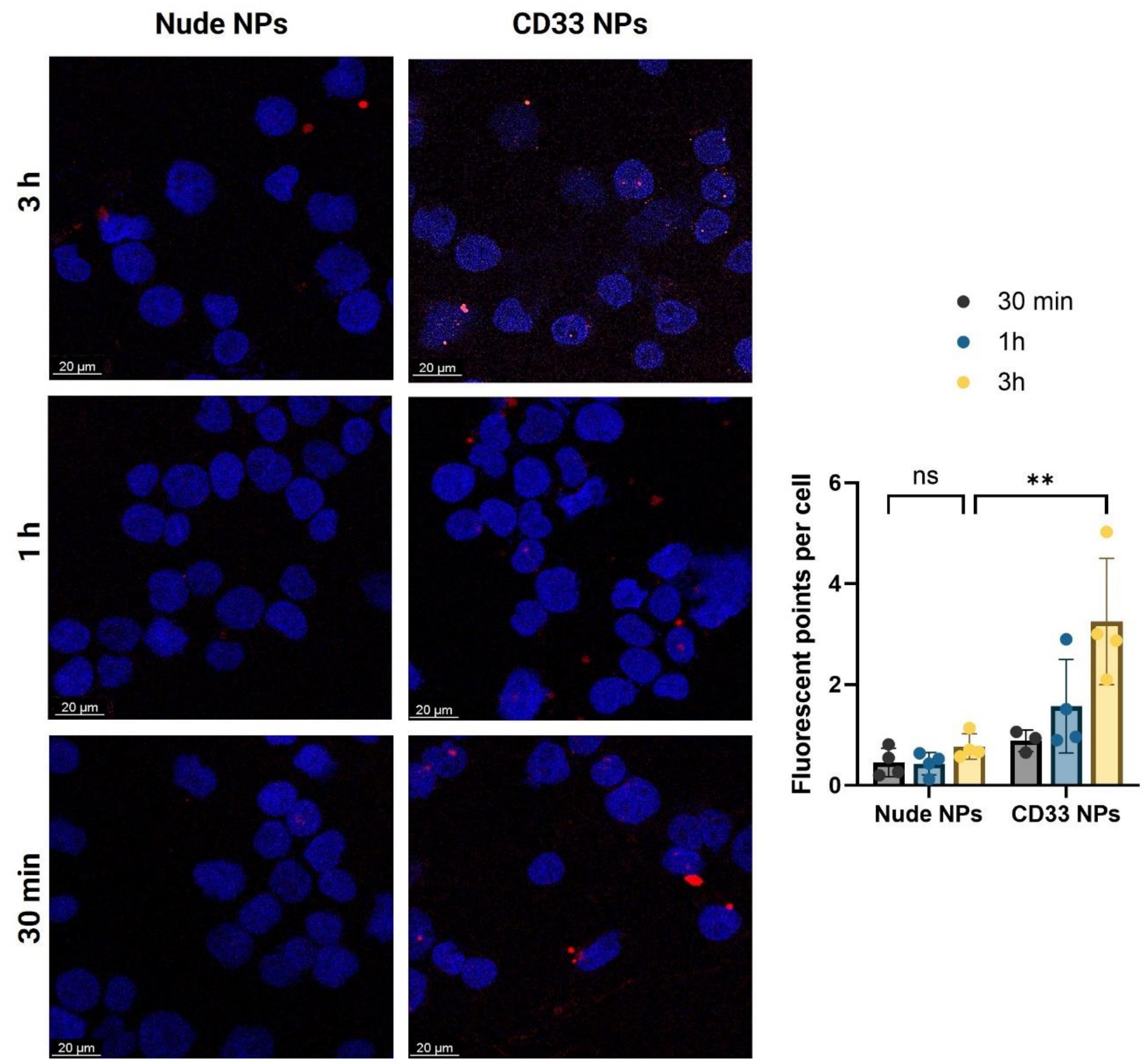

Figure S14. Confocal microscopy images of MOLM-13 cells treated with Rhodamine 6G-loaded NPs in a time course manner. 250 µg polymer/mL was added for 30 min, 1 h, or 3 h at 37ºC to allow internalization prior to cell fixing and microscopy analysis. Blue and red staining denote cell nuclei and nanoparticles, respectively. Bar chart represents the number of fluorescent points in the cell nuclei counted per cell, analyzed from 4 images of 2 independent experiments.
